# Meta-Analysis of RNA-Seq Datasets Identifies Novel Players in Glioblastoma

**DOI:** 10.3390/cancers14235788

**Published:** 2022-11-24

**Authors:** Magy Sallam, Mohamed Mysara, Sarah Baatout, Pieter-Jan Guns, Raghda Ramadan, Mohammed Abderrafi Benotmane

**Affiliations:** 1 Radiobiology Unit, Interdisciplinary Biosciences, Belgian Nuclear Research Centre, SCK CEN, 2400 Mol, Belgium; 2 Laboratory of Physiopharmacology, University of Antwerp, 2610 Wilrijk, Belgium; 3 Department of Molecular Biotechnology, Ghent University, 9000 Ghent, Belgium

**Keywords:** glioblastoma, meta-analysis, ferroptosis, noncoding RNA, lncRNAs, miRNA

## Abstract

**Simple Summary:**

Glioblastoma is a grade IV glioma of heterogeneous nature, which complicates disease pathophysiology and biomarker research. The aim of this meta-analysis was to identify long non-coding RNAs (lncRNAs) and protein-coding genes (PCGs) that are differentially expressed in glioblastoma. Additionally, small RNA-seq of glioblastoma tissues was performed to identify differentially expressed microRNAs (miRNAs) compared to normal tissue controls. The meta-analysis identified 98 and 360 differentially expressed lncRNAs (DElncRNAs) and PCGs (DEPCGs), respectively, in addition to five differentially expressed miRNAs (DEmiRNAs) identified by small RNA-seq. Co-expression correlation network clustering of DElncRNAs/DEPCGs identified a functionally relevant sub-cluster containing DANCR and SNHG6, with DElncRNAs overlapping with TCGA-GBM output. Analysis of the pathways associated with these DElncRNAs and DEPCGs revealed an association with a novel cell death pathway, ferroptosis. Thus, our results confirm the involvement of ferroptosis in glioblastoma pathophysiology and present several candidates for further research

**Abstract:**

Glioblastoma is a devastating grade IV glioma with poor prognosis. Identification of predictive molecular biomarkers of disease progression would substantially contribute to better disease management. In the current study, we performed a meta-analysis of different RNA-seq datasets to identify differentially expressed protein-coding genes (PCGs) and long non-coding RNAs (lncRNAs). This meta-analysis aimed to improve power and reproducibility of the individual studies while identifying overlapping disease-relevant pathways. We supplemented the meta-analysis with small RNA-seq on glioblastoma tissue samples to provide an overall transcriptomic view of glioblastoma. Co-expression correlation of filtered differentially expressed PCGs and lncRNAs identified a functionally relevant sub-cluster containing DANCR and SNHG6, with two novel lncRNAs and two novel PCGs. Small RNA-seq of glioblastoma tissues identified five differentially expressed microRNAs of which three interacted with the functionally relevant sub-cluster. Pathway analysis of this sub-cluster identified several glioblastoma-linked pathways, which were also previously associated with the novel cell death pathway, ferroptosis. In conclusion, the current meta-analysis strengthens evidence of an overarching involvement of ferroptosis in glioblastoma pathogenesis and also suggests some candidates for further analyses.

## 1. Introduction

Glioblastoma is the most common primary brain cancer of glial origin [1,2]. While considered the most aggressive grade of gliomas (grade IV), the etiology of glioblastoma remains largely unclear [3]. Conventional treatment modalities for newly diagnosed glioblastoma patients include surgery with adjunctive radiotherapy and chemotherapy (e.g., temozolomide) [4]. Despite these modalities, the median patient survival for glioblastoma is less than 14 months [5]. Unfortunately, glioblastoma tumors exhibit substantial genetic, epigenetic and transcriptional heterogeneity which adds to the challenge of early diagnosis and therapy development [6]. Recently, non-coding RNAs such as long non-coding RNAs (lncRNAs) and microRNAs (miRNAs) have been associated with different aspects of glioblastoma pathogenesis such as tumorigenesis, proliferation, invasiveness, drug resistance and survival [7,8]. LncRNAs are non-coding RNA transcripts of sizes larger than 200 nucleotides [9]. They regulate gene expression by acting as transcription factor and chromatin modifier guides, molecular scaffolds for enzymatic complexes, and decoy inhibitors of RNA-binding proteins, transcription factors and miRNAs [10,11]. On the other hand, miRNAs are a species of short non-coding RNAs (18–25 nucleotides) which regulate gene expression by binding to mRNAs’ untranslated regions and mediating mRNA decay [12]. Consequently, examination of the interaction between these non-coding RNAs and coding mRNAs could reveal novel disease pathways.

**Figure 1:**
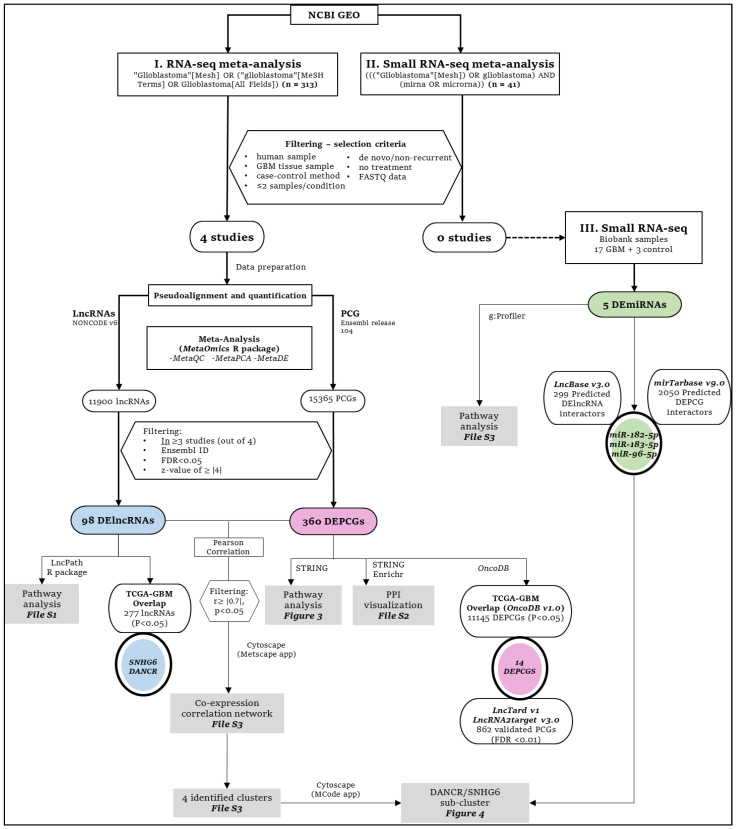
Schematic flow chart of the methodology used in this study. The workflows for lncRNAs, PCGs and miRNAs are denoted via blue, pink and green colors, respectively. Black circles indicate intersection/overlap output with databases. (**I** and **II**) Employed methodology for meta-analysis of glioblastoma tissue RNA-seq and small RNA-seq datasets, respectively. Four studies were selected for RNA-seq meta-analysis with identification of DElncRNAs and DEPCGs, and their overlap with experimentally verified databases and TCGA-GBM. No qualifying studies could be included in small RNA-seq meta-analysis and thus small RNA-seq (**III**) was performed on glioblastoma tissues (n = 17) and normal tissue controls (n = 3) for identification of DEmiRNAs and overlap with predicted miRNA targets of DElncRNAs and DEPCG. Downstream analyses performed on the filtered DElncRNAs, DEPCGs and DEmiRNAs are detailed further with corresponding figures/supplementary files including pathway analyses, protein-protein interactions (PPIs) and co-expression correlation.

Transcriptome research using RNA-seq is regularly used to investigate novel coding and noncoding disease biomarkers, leading to the creation of public databases containing published omics data [13,14,15]. As such, meta-analyses aim to combine this raw data from multiple studies to improve power, accuracy and reproducibility of individual studies [16]. In the current study, we performed a meta-analysis of glioblastoma RNA-seq datasets with differentially expressed protein-coding genes (PCGs) and long non-coding RNAs (lncRNAs), while investigating differentially expressed microRNAs (miRNAs) in glioblastoma tissue samples and normal tissue controls by small RNA-seq. We also identified the overlap between meta-analysis identified PCG/lncRNAs with those identified in The Cancer Genome Atlas Glioblastoma (TCGA-GBM) cohort. Thus, we conducted a transcriptomic examination of de novo/non-recurrent glioblastoma with the aim of identifying novel involvements/pathways. A schematic overview of the methodology employed in our study is shown in Figure 1.

## 2. Materials and Methods

### 2.1. RNA-Seq and Small RNA-Seq Study Selection for Meta-Analysis

We searched glioblastoma-related RNA-seq datasets in GEO DataSet [17] using the following search terms: (“Glioblastoma”[Mesh] OR (“glioblastoma”[MeSH Terms] OR Glioblastoma[All Fields])). The search was performed on 1 February 2020. Filters were applied to show only studies containing expression profiling by high-throughput sequencing or non-coding RNA profiling by high-throughput sequencing. Thus, we selected the suitable datasets using the following criteria: (1) the study was performed in humans; (2) the study in the dataset was designed using the case-control method; (3) the study presented at least two samples per condition (case and control); (4) the assayed samples were sampled from de novo or non-recurrent glioblastoma tumor tissues; (5) the study participants/samples had not received any treatments (radio/chemotherapy); (6) the dataset provided the FASTQ data. Finally, the studies from these datasets were selected (Figure 1). The clinical information of glioblastoma patients and their controls have been reported in the individual studies: study 1 [18], study 2 [19], study 3 [20] and study 4 [21]. From these studies, only glioblastoma and paired control samples were included in our meta-analysis.

For small RNA-seq meta-analysis, similar filtering criteria as those employed in the glioblastoma RNA-seq meta-analysis were applied while searching for glioblastoma-related small RNA-seq datasets in the GEO database. This search was performed on 22 March 2022. The following search terms were employed in our search: ((“Glioblastoma”[Mesh]) OR glioblastoma AND (mirna OR microrna)) while selecting filters for studies performed in humans and containing expression profiling by high-throughput sequencing or non-coding RNA profiling by high-throughput sequencing. Then, the suitable datasets were selected using the same criteria used for RNA-seq meta-analysis.

### 2.2. Quantification of Long Non-Coding RNA (lncRNA) and Protein Coding Gene (PCG) Sequencing Abundance Using RNA-Seq Data

The sequencing data of the selected studies was downloaded by *Prefetch* and converted into FASTQ files using the *fastq-dump* tool of the SRA Toolkit software v2.11.0 [22,23]. Then, the reference sequences of lncRNA and protein-coding transcripts were downloaded from the most complete annotated non-coding RNA databases, NONCODE (v6; [24]), for lncRNAs and Ensembl for PCGs (release 104; [25]), respectively. After merging the two FASTA format files, 199,240 transcript sequences of 173,112 human lncRNA genes were obtained from NONCODE. After removing the pseudogenes, quantification of the lncRNAs and protein-coding genes was performed simultaneously by mapping the RNA-seq reads of each study to the merged reference sequence (pseudoalignment) and calculating the count values using *Kallisto* software v0.46.2 [26]. In addition to the default parameter settings, the estimated average fragment length and the standard deviation of fragment length were set to 200 and 20, respectively. Based on the annotation file *Transcript2Gene*, transcript-level count values of lncRNAs were integrated using the R package *tximport* v1.24.0 to calculate their corresponding gene-level count values.

Quality control was performed using the MetaQC module in the transcriptomic meta-analysis R package *MetaOmics*, and the standardized mean difference (SMD) with its 95% confidence interval (CI) was calculated. For dimension reduction, the MetaPCA module was applied in *MetaOmics* to perform a meta-analytic approach of the principal component analysis (PCA) algorithm of the four selected studies. To identify the significantly differentially expressed lncRNAs and PCGs in glioblastoma tissues, the individual results of each study were integrated by meta-analysis using the MetaDE module of *MetaOmics* for the four selected studies. The normalization process used in this meta-analysis was performed using a random-effects model (REM) for lncRNAs/PCGs with count ≥ 10 [23,27,28]. Differentially expressed lncRNAs (DElncRNA) and differentially expressed PCGs (DEPCGs) were then identified by selecting for lncRNAs/PCGs differentially expressed in at least three studies (out of four), having valid Ensembl ID with FDR < 0.05 and having a z-value of ≥|4|.

### 2.3. Identification of Overlap between DElncRNAs/DEPCGs and lncRNAs/PCGs in Publicly Available Experimentally Verified Databases and TCGA-GBM Output

To further validate the DElncRNAs, a manual search of experimentally validated PCG targets of DElncRNAs was performed by searching in two databases using the Ensembl lncRNA ID: LncTarD v1 [29] and LncRNA2Target v3.0 [30]. For RNA-seq/microarray experiments, targets were selected to have adjusted *p* values < 0.01. In case listed targets had an adjusted *p* < 0.01, all listed targets were selected. After the manual search, overlap between DEPCGs and search-identified PCGs was recorded.

Finally, we investigated the overlap of DElncRNAs and DEPCGs from our meta-analysis with those identified from the TCGA-GBM database, as supplied by LncTard v1 and OncoDB v1.0 (oncodb.org), respectively [29,31]. In LncTard, differential expression patterns of lncRNAs in the TCGA pan-cancer dataset were downloaded and only the expression patterns of the TCGA-GBM cohort were considered. Furthermore, output was filtered according to adjusted *p* value < 0.01. TCGA-GBM expression data were downloaded from the data download portal of OncoDB wherein log_2_ fold change values of tumor and matched normal (control) RNA-seq data had been calculated [31]. Gene overlap between DEPCGs and TCGA-GBM PCGs was then recorded (Figure 1).

### 2.4. Pathway Analysis of DElncRNAs and DEPCGs

The LncRNAs2Pathways R package *LncPath* v1.1 was used to identify the functional pathways of supplied lncRNAs, based on identifying the pathways of associated protein-coding genes (PCGs) [32]. Shortly, the Ensembl IDs of the DElncRNAs were queried using the *LncPath* function in the KEGG and Reactome databases [33,34]. Only pathways with FDR < 0.05 were considered significant.

For pathway analysis and protein-protein interactions, DEPCGs were uploaded to STRING v11.5 (Search Tool for the Retrieval of Interacting Genes/Proteins) online public database (https://string-db.org/ (accessed on 3 March 2022 )) [35].

For visualization of the identified DEPCG-enriched pathways, the STRING network produced by analysis of DEPCGs was imported into Cytoscape 3.9.0 [36]. Using the *String app* v1.7.0 in Cytoscape, we imported the PPI network of DEPCGs, performed STRING enrichment and visualized the identified KEGG and Reactome pathways using the *EnrichmentMap* v3.3.3 app with an edge cut-off of 0.4 and *p* < 0.05. To simplify the resultant STRING network, the Molecular Complex Detection (*MCODE* v2.0.0) app was used to detect densely connected regions in networks and thus identify the biggest DEPCG clusters containing ≥ 10 members [37]. The cluster-finding cutoff parameters were as follows: a *p*-value cutoff of 0.05 and an edge (the degree of gene overlap that exists between two gene sets) cutoff of 0.4.

### 2.5. Co-Expression Analysis of DElncRNAs and DEPCGs and Identification of Highly Connected Nodes

Using the normalized counts of DElncRNAs and DEPCGs, a lncRNA-mRNA co-expression network was built to identify the relationships between DElncRNAs and DEPCGs. We filtered DElncRNAs and DEPCGs to build the network according to the Pearson correlation coefficient (r) > |0.7| with *p* < 0.05. Visualization of the DElncRNAs/DEPCGs correlation was performed using the *Metscape* v.3.1.3 app from Cytoscape software v.3.9.0. Highly connected nodes that had ≥10 DElncRNAs/DEPCGs were identified by clustering the co-expression network using *MCODE*.

### 2.6. Small RNA-Seq of Glioblastoma and Control Tissue Samples

Freshly frozen brain tissue samples from patients with glioblastoma (*n* = 17) and tumor-adjacent normal tissue controls (*n* = 3) were collected from the Biobank Antwerp (University Hospital of Antwerp (UZA), Antwerp, Belgium; ID: BE 71030031000) [38]. These tissue samples were residual material collected within the opt-out system, as stated in the Belgian law of 19 December 2008 whereby residual material may be used for translational research. The study was approved by the local medical ethics committee (Contract number: BB20079).

Total RNA, including microRNAs (miRNAs), was isolated from the glioblastoma tissues and normal controls using the miRNeasy Serum/Plasma kit (Qiagen, Hilden, Germany) according to the manufacturer’s protocol. Total RNA was eluted in a volume of 30 μL RNase-free water. Concentration, purity and integrity of the RNA were determined by spectrophotometry (Little Lunatic, Unchained labs, CA, USA) and the Agilent 2100 Bioanalyzer/Agilent RNA 6000 Nano Kit (Agilent, CA, USA). Library preparation for small RNA-seq and sequencing on Illumina HiSeq of total RNA was performed by GENEWIZ Inc (GENEWIZ, NJ, USA).

Functional enrichment of the identified differentially expressed miRNAs (DEmiRNAs) was performed by importing the Ensembl IDs (ENSG00000283203, ENSG00000207990, ENSG00000207691, ENSG00000208003, and ENSG00000199158 for miR-1246, miR-182, miR-183, miR-549a and miR-96, respectively) into g:Profiler [39]. G: Profiler is a web server offering Gene Ontology (GO) and pathway enrichment analysis resulting from mining high-throughput genomic data [40].

### 2.7. Prediction of Interacting miRNAs of DElncRNAs and DEPCGs in Publicly Available Experimentally Verified Databases

DElncRNA-interacting miRNAs were investigated by supplying our DElncRNAs list into DIANA-LncBase v3.0, which provides a free repository of experimentally supported miRNA targets of lncRNAs [41]. DEPCG-interacting miRNAs were investigated by supplying our DEPCG list into mirTarBase v9.0, which provides the most current miRNA–target interactions by comparisons with other similar databases, such as TarBase, miRecords and miR2Disease [42,43]. Overlap between database-identified interacting miRNAs and differentially expressed miRNAs in glioblastoma tissue samples was identified (Figure 1).

## 3. Results

### 3.1. Four Glioblastoma RNA-Seq Datasets Were Selected for Meta-Analysis

**Figure 2:**
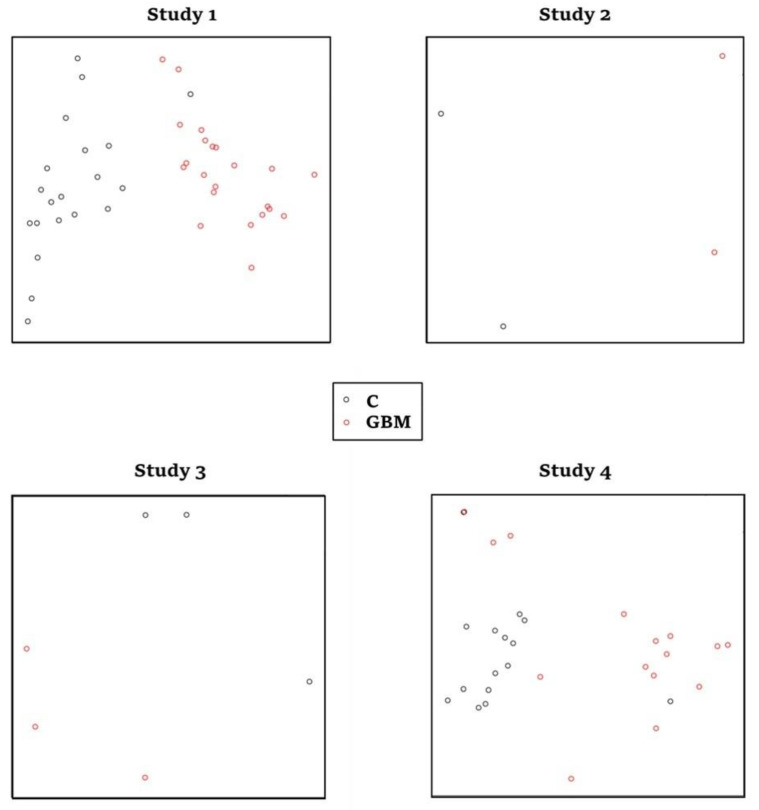
Output from the MetaPCA analytical module of the MetaOmics package showing principal component analyses (PCA) plots for the four selected studies (C: normal tissue controls, GBM: glioblastoma).

Using keyword search and quality filtering, we identified four glioblastoma tissue-related RNA-seq datasets, including: GSE59612, GSE62731, GSE86202 and GSE165595. From the two-dimensional PCA plots of the four selected studies (Figure 2), little variation was found between the glioblastoma tissue samples in each study; however, distinct variation from controls was revealed. After examining the quality control parameters calculated by the MetaQC module, which included internal quality control (IQC), accuracy quality control of gene (AQCg), consistency quality control of gene (CQCg) and standardized mean rank (SMR), no studies were excluded from our analysis.

After analyzing the homogenized data using the bias-resilient random-effects model (REM), LncRNA abundance was quantified in the 84 samples from the four selected studies. In total, 11,900 lncRNAs and 15,365 PCGs were identified from REM meta-analysis. We further limited our downstream validation by selecting lncRNAs differentially expressed in at least three studies (out of four), having Ensembl ID, FDR < 0.05 and a z-value (weighted effect size) of ≥|4|. Consequently, we identified 98 DElncRNAs and 360 DEPCGs fulfilling these criteria. Details of the selected datasets can be found in Table 1; the full selection steps and identified DElncRNAs and DEPCGs are detailed in Supplementary file S1.

### 3.2. Two DElncRNAs (DANCR and SNHG6) and 222 DEPCGs Were also Differentially Expressed in the TCGA-GBM Cohort

Overlap between the list of DElncRNAs and DEPCGs with the TCGA-GBM cohort identified two DElncRNAs (DANCR and SNHG6) and 222 DEPCGs (detailed list available in Supplementary file S1).

Of these 222 DEPCGs, 14 were identified as experimentally validated targets of DANCR during our manual search of experimental databases LncTarD and LncRNA2target (ROCK1, ZWILCH, RPGR, GK, ZNF460, METAP2, CIP2A, ASAH1, ZNF528, C5orf15, QTRT2, STX2, MAP3K2 and CNTRL). Literature-based functionality of these 14 DEPCGs showed that several of these were previously implicated in glioblastoma pathogenesis (Supplementary file S3).

### 3.3. Pathway Analysis of DElncRNAs Reveals Several Glioblastoma-Associated Pathways

Pathway analysis identified four KEGG and 37 Reactome significantly enriched pathways (FDR < 0.05) that were associated with DElncRNAs (Supplementary file S1). The top pathways according to the normalized enrichment scores were glycoprotein-related pathways (O-glycan biosynthesis, O-linked glycosylation of mucins, termination of O-glycan biosynthesis and HS-GAG degradation of glycoprotein), the Fanconi anemia pathway, the glutamate neurotransmitter release cycle, interaction between L1 and ankyrins and SRP-dependent cotranslational protein targeting to membrane, which have been previously associated with glioblastoma [44,45]

### 3.4. DEPCGs Show a Highly Connected PPI Network with Several Enriched Glioblastoma-Linked Pathways

**Figure 3:**
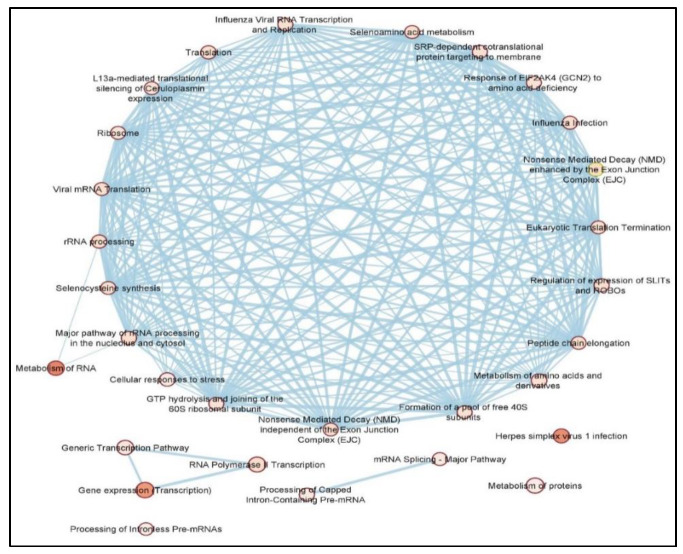
Enrichment map of KEGG (dark pink nodes) and Reactome (light pink nodes) pathways of DEPCGs as indicated by STRING enrichment in Cytoscape. The thickness of a line indicates the strength of the interaction between the proteins it connects.

Analysis of DEPCGs using STRING databases produced a highly connected protein-protein interaction network (PPI) (Supplementary file S2). Functional enrichment of the produced PPI network identified a number of significantly enriched KEGG and Reactome pathways (FDR < 0.05) (Figure 3) e.g., nonsense-mediated decay (NMD), L13a-mediated silencing of ceruloplasmin expression, EIF2AK4 response to amino acid deprivation, regulation of expression of SLITs and ROBOs and selenocysteine synthesis.

Clustering of the PPI network into individual clusters containing ≥ 10 DEPCGs yielded only one cluster that showed nearly identical functional enrichment as the parent PPI.

Pathway enrichment overlap between DEPCGs and DElncRNAs revealed several overlapping pathways (Table 2). From these, NMD and SRP-dependent cotranslational protein targeting to membrane have been associated with glioblastoma. However, others, such as influenza viral RNA transcription and replication, have not been directly associated with glioblastoma.

### 3.5. Three DEmiRNAs Identified by Small RNA-Seq of Glioblastoma Tissue Overlap with Predicted DElncRNA and DEPCGs-Interacting miRNAs

The glioblastoma-related small RNA-seq dataset search yielded 41 datasets. After applying filtering criteria, none of these datasets qualified for inclusion in our analyses. Search results and rejection criteria are detailed in Supplementary file S1. Subsequently, analysis of small RNA-seq of glioblastoma tumor tissue and controls identified several differentially expressed miRNAs, of which five were significantly differentially expressed miRNAs (DEmiRNAs): hsa-miR-1246, hsa-miR-182-5p, hsa-miR-183 (-3p and -5p), hsa-miR-549a and hsa-miR-96-5p (*p* < 0.05). Functional enrichment of DEmiRNAs identified an enrichment in several GO: Biological Processes, which were all associated with the traditional miRNA roles in post-transcriptional regulation as well as enrichment of the KEGG pathway “MiRNAs in cancer” (Supplementary file S3).

From mirTarBase, 2050 unique miRNAs were identified as interacting miRNAs of DEPCGs by one of the following methods: reporter assay, western blot, qPCR, microarray, pSILAC, NGS, other validation methods or CLIP-Seq. From LncBase, 299 unique miRNAs were identified as interacting miRNAs of DElncRNAs. Detailed output of mirTarBase and LncBase search results of DEPCGs and DElncRNAs, respectively, can be found in Supplementary file S1.

Overlap between DEmiRNAs and predicted interacting miRNAs of DElncRNAs and DEPCGs identified three miRNAs: hsa-miR-182-5p, hsa-miR-183 (-3p and -5p) and hsa-miR-96-5p, which were previously identified as experimentally validated targets of DANCR and SNHG6.

### 3.6. Co-Expression Analysis Identifies 4 Clusters of DElncRNAs/DEPCGs

Analysis of co-expression of DElncRNA and DEPCGs revealed 15731 correlation pairs having r ≥ |0.7| and *p* < 0.05 supplied in Supplementary file S1. Clustering of the network using MCODE default settings into clusters containing ≥10 members yielded four individual clusters of which the first cluster was further clustered into three main sub-clusters (Supplementary file S3).

**Figure 4:**
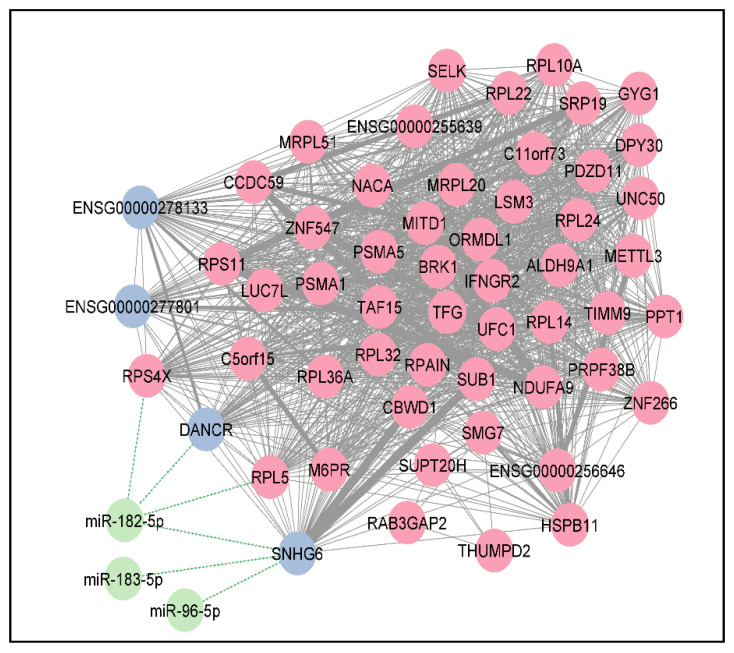
The DANCR/SNHG6 sub-cluster of DElncRNA-DEPCG (blue and pink circles, respectively) co-expression correlation network produced was visualized using MCODE in Cytoscape, supplemented with interacting DEmiRNAs (green circles) as supplied by mirTarBase and LncBase databases. STRING enrichment analysis of this sub-cluster shows strong similarity with DEPCG enrichment, thereby denoting sub-cluster relevance.

Pathway analysis of the individual clusters and sub-clusters revealed that only one sub-cluster (Figure 4) (containing DANCR and SNHG6) was responsible for the majority of the enriched pathway associations identified for DEPCGs and DElncRNAs, e.g., L13a-mediated silencing of ceruloplasmin expression, regulation of expression of SLITs and ROBOs and selenocysteine synthesis, EIF2AK4 response to amino acid deprivation and NMD (Supplementary file S1).

## 4. Discussion

Previous meta-analyses have elucidated previously unexamined relevance to specific pathways as well as aided in the identification of candidate biomarkers [46,47]. Meta-analyses have advantages over single studies of effect consistency with enhanced statistical power [48]. Therefore, we performed a meta-analysis of publicly available RNA-seq glioblastoma datasets of non-recurrent glioblastoma and control samples from the same patient. In this manner, 98 DElncRNAs and 360 DEPCGs were identified. We also performed small RNA-seq of glioblastoma tissues and normal controls.

### 4.1. Meta-Analysis of Glioblastoma RNA-Seq Datasets

#### 4.1.1. DElncRNAs

The top five identified DElncRNAs according to absolute weighted-effect size included four DElncRNAs that had no previously characterized roles in glioblastoma; RNFT1-DT, ENSG00000233184, ENSG00000268205 and ENSG00000268362, as well as glioblastoma prognostic biomarker MROCKI (LINC01268) [49]. Due to the high differential expression of these DElncRNAs, future studies determining their specific roles in glioblastoma could reveal novel involvements.

Functional enrichment of the full 98 DElncRNAs revealed over 30 significantly enriched pathways previously identified in glioma, including pathways associated with O-glycans (O-glycan biosynthesis, O-linked glycosylation of mucins, termination of O-glycan biosynthesis and HS-GAG degradation of glycoproteins), the Fanconi anemia pathway, the glutamate neurotransmitter release cycle, insulin receptor recycling, interaction between L1 and ankyrins as well as transferrin endocytosis and recycling (Supplementary file S1). O-glycans are found on glycoproteins, of which mucins are the main class, which regulate protein folding, stability and trafficking, and also mediate many cell-cell interactions [50,51]. Many cancers express altered mucin-type O-glycans (reviewed in [52]) including glioma where aberrant glycosylation of tumor glycan-rich extracellular matrix promotes tumor progression and treatment resistance [44]. On the other hand, the Fanconi anemia (FA) pathway relates to DNA damage repair processes of lesions in the replication fork which impede replication [53]. This pathway is reactivated in glioblastoma, mediating survival of the mutated cells and thereby accelerating carcinogenesis [45,53]. Alternately, glutamate is produced in glioma cells as a byproduct of glutathione synthesis, leading to tumor expansion and invasion [54,55]. Insulin receptor recycling frees insulin receptors to engage in downstream signaling regulating cell proliferation, which worsens glioblastoma prognosis and mediates treatment resistance [56]. L1 cell adhesion molecule (L1cam) is a neural adhesion molecule whose levels have been shown to associate with glioblastoma, and its knockdown can suppress glioma stem cell growth [57,58]. Finally, transferrin is a glycoprotein responsible for iron ion delivery that is overexpressed in glioblastoma, leading to increased cell proliferation and worsening prognosis [59].

#### 4.1.2. DEPCGs

Similarly, the top five DEPCGs according to weighted effect size included ATF6, AHCTF1, ZCCHC10, ZNF234 and IFNGR2. Of these, only ATF6 and IFNGR2 have been previously associated with glioblastoma viability and treatment resistance, while the remaining three have only been identified in other cancer types, which encourages further investigations [60,61,62,63,64,65]. Moreover, several significantly enriched pathways were identified by pathway-enrichment analysis of the 360 DEPCGs (Figure 3), such as nonsense-mediated decay (NMD), ceruloplasmin expression, selenocysteine synthesis, SLIT/ROBO signaling, as well as EIF2AK4 and Hedgehog signaling. NMD functions to eliminate truncated mRNA transcripts resulting from premature termination codons (PTCs), protecting against their dominant negative effect on the functional wild-type alleles [66]. Inhibition of NMD regulates tumorigenesis and stemness properties in glioma stem cells [67]. Ceruloplasmin is a copper-binding protein which regulates iron efflux [68]. In glioblastoma, ceruloplasmin leads to excessive extracellular iron with subsequent oxidative stress, impacting blood-brain barrier integrity [69]. Another enriched pathway was synthesis of selenocysteine which is a selenium containing amino acid incorporated in anti-oxidant selenoproteins, such as glutathione peroxidases, and has been shown to induce apoptosis of glioblastoma cells in vitro [70,71]. On the other hand, Slits (ligands) and Robos (receptors) are glycoproteins involved in several cell signaling pathways including axon guidance, cell proliferation, cell motility and angiogenesis (reviewed in [72]). The effects of Slit/Robo signaling in glioblastoma are not clearly characterized. On the one hand, Slit2 expression is suppressed in glioma cells and intracranial mice xenografts with forced expression hampering glioma cell migration and invasion [73]. On the other hand, Slit2 knockdown in mouse glioma cells and patient-derived GBM xenografts decreased tumor growth and increased treatment resistance [74]. In either case, Slit2 levels seem to influence glioblastoma growth and treatment resistance; however, further research is needed to elucidate its exact role. Alternately, EIF2AK4, eukaryotic translation initiation factor 2 alpha kinase 4, is activated by metabolic stress signals to induce global protein translation inhibition and cell survival control [75]. Normally, as tumor growth progresses, access to nutrients such as amino acids decreases, which activates EIF2AK4 to induce downstream effects of increased tumor cell survival and treatment resistance [76,77]. This was shown in our pathway analysis by the identification of the involvement of amino acid metabolism and peptide chain elongation pathways. Finally, the Hedgehog pathway is essential during development for intercellular communication, organogenesis, regeneration and homeostasis [78]. The exact mechanisms of Hedgehog pathway tumorigenic activity are reviewed in [79,80]. In glioblastoma, Hedgehog pathway inhibitors were shown to decrease cancer stem cell growth and drug resistance [81,82].

### 4.2. Small RNA-Seq of Glioblastoma Tissues and Normal Controls

In the current study, small RNA-seq identified five differentially expressed microRNAs (DEmiRNAs): miR-1246, miR-182-5p, miR-183 (-3p and -5p), miR-549a and miR-96-5p. Functional enrichment of these DEmiRNAs was uninformative. However, each of these five DEmiRNAs has been previously identified in glioblastoma. Increased exosomal miR-1246 expression was found to promote a pro-oncogenic immunosuppressive microenvironment, while it was associated with a higher glioma recurrence rate in postoperative patients [83]. Previous studies also linked miR-182-5p to glioblastoma tumorigenesis, angiogenesis and metastasis [84,85]. Alternatively, mir-183 is a TGFβ-induced miRNA which also contributes to the immunosuppressive glioma microenvironment [86,87]. In fact, miR-183-5p has been proposed to be a prognostic biomarker of glioblastoma progression [88,89]. Similarly, mir-549a was previously shown to be of prognostic importance in tumors of glial origin [90]. Finally, miR- 96-5p was found to be upregulated in glioma cells, with effects on proliferation and metastasis [91]. Upregulation of miR-96 was also found to promote radioresistance in T98G glioblastoma cells [92]. Interestingly, miR-182, miR-183 and miR-96 are located within less than 4.5 kbp of one another and comprise the miR-183/96/182 cluster [87]. This miR-183/96/182 cluster was associated with the progression from low- to high-grade glioma (glioblastoma) while knockdown of this cluster in glioblastoma inhibited cell survival [87,88].

### 4.3. Overlap with Glioblastoma-Relevant Databases

Overlap of our DElncRNA and DEPCGs with The Cancer Genome Atlas glioblastoma (TCGA-GBM) patient cohort output yielded two lncRNAs (DANCR and SNHG6) and 222 DEPCGs (Supplementary file S1). DANCR is an oncogenic lncRNA which induces several cancer-promoting effects, such as promotion of angiogenesis and epigenetic silencing of tumor-suppressors; it also regulates cancer-promoting signaling pathways such as the Wnt/β-catenin, JAK/STAT, Notch, and PI3K/AKT pathways (reviewed in [93]). Due to its pan-oncogenic effect, DANCR has been considered to be a candidate cancer therapeutic target [94,95]. In glioma, DANCR knockdown leads to decreased proliferation and migration [96]. The oncogenic effects of DANCR seem to be caused mainly by its role as a competing endogenous RNA (ceRNA), which binds miRNAs competitively, thereby influencing miRNA capacity to inhibit mRNA translation [95]. In glioma cells, DANCR was shown to act as ceRNA to miR-634, a miRNA shown to increase glioma cell sensitivity to temozolomide [97,98]. DANCR was also shown to promote cisplatin resistance via ceRNA-mediated inhibition of sponging miR-33a-5p, miR-33b-5p, miR-1-3p, miR-206, and miR-613 with resultant activation of the AXL/PI3K/Akt/NF-κB signaling pathway [99].

Similarly to DANCR, SNHG6 was shown to promote glioma progression via a similar ceRNA activity by interfering with glioma-relevant miRNAs: miR-543 and miR-101-3p [100,101]. SNHG6 was also shown to promote glioma malignant progression by inducing histone modifications in tumor suppressor genes [102].

Of the 222 DEPCG overlapping with TCGA-GBM, 14 were identified to be DANCR-regulated by searching of LncRNA2Target and LncTard databases. Literature-based functionality of these 14 DEPCGs showed that several of them were previously implicated in glioblastoma proliferation, invasiveness and treatment resistance (Supplementary file S3), thereby explaining some of the pro-tumorigenic effects of DANCR. For the remaining seven DEPCGs (ZWILCH, RPGR, ZNF460, ZNF528, QTRT2, C5orf15 and CNTRL), no previous functional associations were found with glioblastoma progression, despite a number of them being associated with other cancer types [103,104,105,106,107]. Future investigations into potential previously unaddressed roles of these genes could reveal new players in glioblastoma pathogenesis.

Due to the study selection process and applied filtering criteria, the data from the Ivy Glioblastoma Atlas (IVY GAP) [108] and Chinese Glioma Genome Atlas (CGGA) [109] were not included in our assays (both not being in case-control format). However, in CGGA, the co-expression correlation between the two TCGA-GBM overlapping DElncRNAs (DANCR and SNHG6) and three DEmiRNAs (miR-96, miR-182 and miR-183) was assayed through the ‘Analyze’ portal on the CGGA website (http://www.cgga.org.cn/, accessed on 22 November 2022). In the CGGA RNA-seq datasets, DANCR expression showed a significant medium correlation to SNHG6 expression (R = 0.446 and 0.449 for dataset mRNAseq_693 and mRNAseq_325, respectively, *p* < 0.001 for both) (Supplementary file S3). Using the CGGA miRNA array dataset, a significant strong correlation was identified between the three miRNAs (miR96/miR182: R = 0.721, *p* < 0.001, miR96/miR183: R = 0.745, *p* < 0.001 and miR182/miR183: R = 0.937, *p* < 0.001) which is unsurprising as they form the miR-183/96/182 cluster (Supplementary file S3). This confirms the strong interaction between DANCR and SNHG6, as well as between the DEmiRNAs in the miR-183/96/182 cluster in CGGA, as was replicated by our analyses.

### 4.4. Co-Expression Correlation Network Construction and Functional Enrichment

A co-expression network was also constructed to identify DElncRNA/DEPCG highly interacting pairs with possible functional associations. A strong correlation was found between DANCR and SNHG6 expression (r = 0.76 and *p* < 0.001), which confirms the similar correlation observed in CGGA datasets.

In addition, clustering of the co-expression network and pathway analysis of the identified clusters and sub-clusters revealed that the sub-cluster containing DANCR and SNHG6 was responsible for a majority of the pathway enrichments of the 360 DEPCGs. Interestingly, both DANCR and SNHG6 are targets of the miR-183/96/182 cluster in the DIANA-LncBase database, which suggests a possible DElncRNA/DEmiRNA interplay in glioblastoma. In addition, two novel DElncRNAs (ENSG00000278133 and ENSG00000277801) were found to belong to this cluster. The high degree of interactions between these two DElncRNAs with the DEPCGs sub-cluster members suggests a possible novel relevance in glioblastoma, thereby necessitating future research.

Seven DEPCGs in the DANCR/SNHG6 sub-cluster were also differentially expressed in TCGA-GBM, while being involved in ≥20 of the identified enriched pathways of the sub-cluster. These genes were ribosomal proteins RPS11, RPL5, RPL10, RPL24, RPL14, RPL36A and RPL32. Only RPS11 and RPL36A were previously found to be beneficial in glioma as prognostic predictors [110,111,112,113,114]. Therefore, it may be useful to examine the exact roles of the remaining unexplored DEPCGs in glioblastoma.

### 4.5. Literature-Based Associations of the Identified Pathways: Deducible Involvement of Ferroptosis?

Literature-based research of the DElncRNA and DEPCG-enriched pathways led to the identification of their shared association with the novel cell death pathway, ferroptosis [115]. Ferroptosis is a recently discovered intracellular iron-dependent form of cell death characterized by the overproduction of reactive oxygen species (ROS) and accumulation of lipid peroxidation, leading to cell death [116]. As glioblastoma cells have higher ROS and iron accumulation than healthy tissues, they are especially susceptible to death by ferroptosis [117,118]. As a result, ferroptosis induction inhibits glioblastoma tumor growth, improves patient survival and increases the efficacy of radio- and chemotherapy, thereby providing adjuvant antitumor options [119,120].

Ferroptosis was shown to be regulated by DElncRNA-enriched pathways, protein O-glycosylation as well as glutamine, glutamate and transferrin [121,122,123,124]. On the other hand, ferroptosis was previously shown to be influenced by DEPCG-enriched pathways, induced by glutathione peroxidase suppression (selenocysteine-containing enzyme) and SMG9 (a component of the NMD machinery) and inhibited by ceruloplasmin and Hedgehog pathway activation [125,126,127,128,129,130,131]. EIF2AK4 was also identified in a ferroptosis-associated gene signature in glioma [132], while concurrent dysregulation of ferroptosis and the SLIT/ROBO signaling pathway has been associated with low-grade endometrial cancer [133]. Consequently, the identified DElncRNAs/DEPCGs seem to suggest an association between glioblastoma and ferroptosis in our analyzed datasets.

As the DANCR/SNHG6 sub-cluster possesses similar enrichments to the identified DEPCG pathways, we investigated whether these sub-cluster members had identifiable associations with ferroptosis. Both DANCR and SNHG6 were previously associated with ferroptosis [134,135]. Some of the DEPCG members of the sub-cluster have also been shown to regulate ferroptosis (Supplementary file S3). However, the majority of the sub-cluster members had no previous connections to ferroptosis. Consequently, due to the high interaction between this sub-cluster and its enriched pathways, this sub-cluster could identify future candidates for glioblastoma biomarkers or treatment modulators.

To further confirm this connection, we also investigated whether the DEmiRNAs had previous associations with ferroptosis. All DEmiRNAs in the miR-183/96/182 had been previously associated with ferroptotic processes in the literature [136,137,138]. However, the exact involvement of these DEmiRNAs in ferroptosis processes in glioblastoma is currently under-researched. Therefore, future studies could reveal a role for these DEmiRNA in modulation of glioblastoma responsiveness to treatment.

### 4.6. Limitations

Our study does have some limitations. Firstly, only four glioblastoma datasets were included in our analysis, due to the study selection criteria. Quality assessment (MetaQC) of the included studies resulted in the inclusion of all four studies in our analyses, despite the low sample size in certain instances. However, we attempted to overcome this limitation by overlapping our findings with larger glioblastoma datasets, such as TCGA and CGGA. Secondly, glioblastoma can be sub-classified into proneural, neural, mesenchymal, and classical according to differential gene expression profiles, as well as the mutation status of certain key genes including platelet-derived growth factor receptor (PDGFRA), neurofibromatosis type 1 (NF1) and epidermal growth factor receptor (EGFR) [139,140]. Also, recent WHO updates to CNS tumor nomenclature have limited glioblastoma classification to IDH-wildtype adult-type diffuse gliomas [2]. Unfortunately, only two of our included studies contained detailed information about the subclass of the assayed glioblastoma tumors and thus these classifications could not be included in our analyses. We attempted to overcome this heterogeneity by employing a random-effects model (REM), which combines the effect size of the individual studies using a simple linear model with sampling error, while assuming a possible random effect on the effect size of each study [141,142]. However, repeat analyses of previously published datasets after reclassification, according to the current guidelines, could offer novel insights and warrant further research. Thirdly, the limited residual glioblastoma tissue available impacted the number of possible wet-lab validations. Therefore, we recommend the validation of the promising DElncRNA and DEPCG candidates in independent glioblastoma sample cohorts. Finally, our study analyzed RNA-seq of glioblastoma tissues, which involves an invasive sampling procedure that is unsuitable for regular treatment monitoring. Further studies addressing the need for circulating glioblastoma biomarkers are thus of particular interest, with a specific focus on ncRNA due to their relatively higher stability [143,144]. Consequently, further research addressing the usefulness of the identified DElncRNAs and DEmiRNAs as candidate biomarkers and their utilization for routine monitoring is required.

## 5. Conclusions

In this study, we have presented DElncRNAs/DEPCGs which were identified by overlap with a TCGA-GBM cohort and experimental databases, or by inclusion in the most pathway enriched sub-cluster in our co-expression network (also interacting with three of the identified DEmiRNAs). We reviewed the literature for the DElncRNAs/DEPCGs associations with glioblastoma. For some DElncRNAs/DEPCGs, no previous connections to glioblastoma were found, which could provide starting points for future studies. Using literature association of identified DElncRNAs/DEPCGs, we also found a reproducible involvement of ferroptosis. Several identified DElncRNAs, DEPCGs and DEmiRNAs were previously associated with ferroptosis, while the majority still require further investigation. In summary, our study identified a number of candidates for further investigation, while demonstrating a recurring association of ferroptosis with identified glioblastoma pathways.

**Figure 5:**
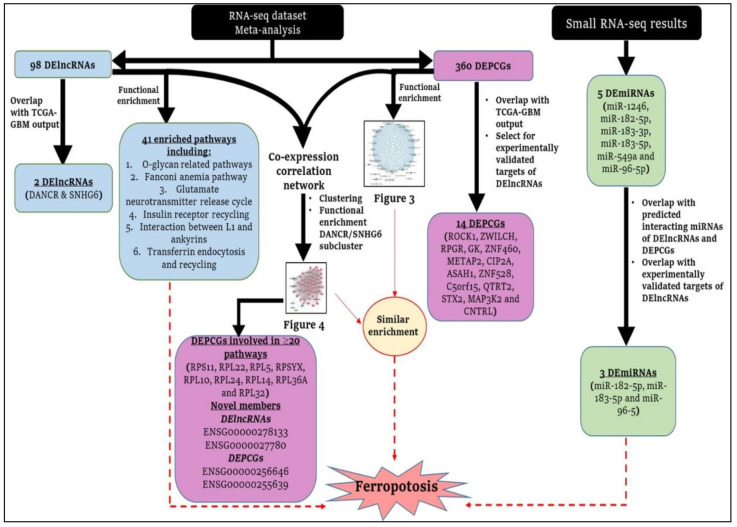
Summary of DElncRNAs (blue), DEPCGs (pink) and DEmiRNAs (green) identified in our study and their connection to ferroptosis.

A summary of the main findings of our study can be found in Figure 5.

## Figures and Tables

**Table 1:** Details of studies fulfilling the predefined criteria with quality control measurements as supplied by the MetaOmics MetaQC module. IQC, internal quality control; AQCg, accuracy quality control of gene; CQCg, consistency quality control of gene; SMR, standardized mean rank.

Study	Dataset	Platform	Sample Size	IQC	AQCg	CQCg	SMR	Reference
1	GSE59612	Illumina HiSeq 2000	22 glioblastoma tumor tissue, 22 controls	5.6	61.25	145.18	1.67	[18]
2	GSE62731	Illumina HiSeq 2000	2 glioblastoma tumor tissue, 2 controls	3.3	2.49	52.78	3.33	[19]
3	GSE86202	Illumina HiSeq 2500	3 glioblastoma tumor tissue, 3 controls	1.3	2.66	17.74	3.67	[20]
4	GSE165595	Illumina HiSeq 4000	15 glioblastoma tumor tissue, 15 controls	5.6	23.08	240.99	1.33	[21]

**Table 2:** Significantly enriched pathways associated with both DElncRNAs and DEPCGs (FDR < 0.05) as identified by LncPath R package and STRING database, respectively.

Database	Overlapping DElncRNA/DEPCGs Associated Pathways (FDR < 0.05)
KEGG	Ribosome
Reactome	Translation Peptide Chain Elongation Influenza Viral RNA Transcription And Replication Nonsense-Mediated Decay Enhanced By The Exon Junction Complex SRP-dependent co-translational protein targeting to membrane

## Data Availability

The small RNA-seq data generated in the study have been submitted to the NCBI Gene Expression Omnibus (GEO) (http://www.ncbi.nlm.nih.gov/geo/) under accession number GSE214252.

## Supplementary Materials

The following supporting information can be downloaded at: https://www.mdpi.com/article/10.3390/cancers14235788/s1, Supplementary file S1: Detailed meta-analysis study selection steps and database/pathway analysis outputs; Supplementary file S2: DEPCG PPI network according to enriched Reactome pathways; Supplementary file S3: Supplementary figures with literature-based evidence of glioblastoma and glioblastoma/ferroptosis functional associations of DANCR-targeted DEPCG overlapping with TCGA-GBM and DEPCGs in the DANCR/SNHG6 sub-cluster, respectively.
