# Supplementary material for: Meta-Analysis of RNA-Seq Datasets Identifies Novel Players in Glioblastoma
Source: Cancers (Basel). 2022 Nov 24;14(23):5788. doi: 10.3390/cancers14235788 (PMC9737249; doi:10.3390/cancers14235788)
Supplement: Supplementary file 1 [file cancers-14-05788-s001.zip › supplementary file S2/DEPCG PPI network according to Reactome pathway legend.pdf]

DEPCG PPI network according to Reactome pathway

Annotation Roadmap:

Reactome Pathway	Associated genes
Metabolism of RNA	AQR|PAPOLA|NOP58|RPS11|PSMA5|UTP15|METTL3|LSM3|WDR75|CSTF3|WDR33|SRSF7|PNRC2|SF3B1|DHX15|RPS17|RPL22|PSMD12|EPRS|TFB1M|RPL5|NDC1|UTP11L|RPS4X|RPL10A|NCBP1|POLR2B|DCP2|RPL24|RPL14|CNOT10|RPL36A|PSMA1|RPL32|DDX46|U2SURP|QTRTD1|WDR36|SMG7|RPL28
Gene expression (Transcription)	PAPOLA|ZNF221|IPO8|SMURF2|POLR1A|ZNF614|PSMA5|ZNF547|BTG2|CSTF3|USP9X|WDR33|MRE11A|SRSF7|ZNF571|POLR3C|ZNF555|ARID2|SF3B1|DPY30|ZNF649|PSMD12|ZNF254|ZNF460|ZNF528|CTR9|INTS7|MDM4|CDC73|RNF2|ZNF75D|NDC1|ZNF684|AGO3|TFAP2E|NCBP1|ZNF549|POLR2B|ZNF566|CDC23|ZNF197|ZNF586|MED13|SSB|CNOT10|ZNF234|GAMT|PSMA1|SKIL|ZNF398|LAMTOR3|DROSHA|INTS8|INTS4|TET2|ZNF266|ZNF253|ZNF2|ZNF227
Nonsense Mediated Decay (NMD) enhanced by the Exon Junction Complex (EJC)	RPS11|PNRC2|RPS17|RPL22|RPL5|RPS4X|RPL10A|NCBP1|RPL24|RPL14|RPL36A|RPL32|SMG7|RPL28
RNA Polymerase II Transcription	PAPOLA|ZNF221|SMURF2|ZNF614|PSMA5|ZNF547|BTG2|CSTF3|USP9X|WDR33|MRE11A|SRSF7|ZNF571|ZNF555|ARID2|DPY30|ZNF649|PSMD12|ZNF254|ZNF460|ZNF528|CTR9|INTS7|MDM4|CDC73|RNF2|ZNF75D|ZNF684|AGO3|TFAP2E|NCBP1|ZNF549|POLR2B|ZNF566|CDC23|ZNF197|ZNF586|MED13|CNOT10|ZNF234|GAMT|PSMA1|SKIL|ZNF398|LAMTOR3|INTS8|INTS4|ZNF266|ZNF253|ZNF2|ZNF227
Influenza Infection	EIF2AK2|RPS11|KPNA4|RPS17|RPL22|RPL5|NDC1|RPS4X|RPL10A|POLR2B|RPL24|RPL14|RPL36A|RPL32|RPL28
Major pathway of rRNA processing in the nucleolus and cytosol	NOP58|RPS11|UTP15|WDR75|RPS17|RPL22|RPL5|UTP11L|RPS4X|RPL10A|RPL24|RPL14|RPL36A|RPL32|WDR36|RPL28
rRNA processing	NOP58|RPS11|UTP15|WDR75|RPS17|RPL22|TFB1M|RPL5|UTP11L|RPS4X|RPL10A|RPL24|RPL14|RPL36A|RPL32|WDR36|RPL28
Nonsense Mediated Decay (NMD) independent of the Exon Junction Complex (EJC)	RPS11|RPS17|RPL22|RPL5|RPS4X|RPL10A|NCBP1|RPL24|RPL14|RPL36A|RPL32|RPL28
Regulation of expression of SLITs and ROBOs	RPS11|PSMA5|RPS17|RPL22|PSMD12|RPL5|RPS4X|RPL10A|NCBP1|RPL24|RPL14|RPL36A|PSMA1|RPL32|RPL28
Peptide chain elongation	RPS11|RPS17|RPL22|RPL5|RPS4X|RPL10A|RPL24|RPL14|RPL36A|RPL32|RPL28
Viral mRNA Translation	RPS11|RPS17|RPL22|RPL5|RPS4X|RPL10A|RPL24|RPL14|RPL36A|RPL32|RPL28

SRP-dependent cotranslational protein targeting to membrane	RPS11|RPS17|RPL22|RPL5|RPS4X|RPL10A|RPL24|RPL14|RPL36A|RPL32|SRP19|RPL28
Influenza Viral RNA Transcription and Replication	RPS11|RPS17|RPL22|RPL5|NDC1|RPS4X|RPL10A|POLR2B|RPL24|RPL14|RPL36A|RPL32|RPL28
Selenoamino acid metabolism	RPS11|RPS17|RPL22|EPRS|RPL5|RPS4X|RPL10A|RPL24|RPL14|RPL36A|RPL32|RPL28
Selenocysteine synthesis	RPS11|RPS17|RPL22|RPL5|RPS4X|RPL10A|RPL24|RPL14|RPL36A|RPL32|RPL28
Eukaryotic Translation Termination	RPS11|RPS17|RPL22|RPL5|RPS4X|RPL10A|RPL24|RPL14|RPL36A|RPL32|RPL28
Translation	MRPL51|RPS11|MRPL39|FARS2|MRPL20|PPA2|RPS17|RPL22|EPRS|RARS2|RPL5|RPS4X|RPL10A|RPL24|RPL14|RPL36A|RPL32|SRP19|RPL28
Formation of a pool of free 40S subunits	RPS11|RPS17|RPL22|RPL5|RPS4X|RPL10A|RPL24|RPL14|RPL36A|RPL32|RPL28
Response of EIF2AK4 (GCN2) to amino acid deficiency	RPS11|RPS17|RPL22|RPL5|RPS4X|RPL10A|RPL24|RPL14|RPL36A|RPL32|RPL28
Metabolism of amino acids and derivatives	RPS11|PSMA5|SLC25A15|RPS17|RPL22|ALDH9A1|PSMD12|RIMKLB|EPRS|RPL5|SARDH|RPS4X|RPL10A|LIPT1|RPL24|RPL14|GAMT|RPL36A|PSMA1|RPL32|RPL28
L13a-mediated translational silencing of Ceruloplasmin expression	RPS11|RPS17|RPL22|RPL5|RPS4X|RPL10A|RPL24|RPL14|RPL36A|RPL32|RPL28
GTP hydrolysis and joining of the 60S ribosomal subunit	RPS11|RPS17|RPL22|RPL5|RPS4X|RPL10A|RPL24|RPL14|RPL36A|RPL32|RPL28
Generic Transcription Pathway	ZNF221|SMURF2|ZNF614|PSMA5|ZNF547|BTG2|USP9X|MRE11A|ZNF571|ZNF555|ARID2|DPY30|ZNF649|PSMD12|ZNF254|ZNF460|ZNF528|MDM4|RNF2|ZNF75D|ZNF684|AGO3|TFAP2E|ZNF549|POLR2B|ZNF566|CDC23|ZNF197|ZNF586|MED13|CNOT10|ZNF234|GAMT|PSMA1|SKIL|ZNF398|LAMTOR3|ZNF266|ZNF253|ZNF2|ZNF227
mRNA Splicing - Major Pathway	AQR|PAPOLA|LSM3|CSTF3|WDR33|SRSF7|SF3B1|DHX15|NCBP1|POLR2B|DDX46|U2SURP
Processing of Capped Intron-Containing Pre-mRNA	AQR|PAPOLA|METTL3|LSM3|CSTF3|WDR33|SRSF7|SF3B1|DHX15|NDC1|NCBP1|POLR2B|DDX46|U2SURP
Cellular responses to stress	RPS11|PSMA5|C11orf73|MRE11A|RPS17|RPL22|PSMD12|MDM4|RNF2|RPL5|NDC1|AGO3|RPS4X|RPL10A|RPL24|CDC23|RPL14|RPL36A|PSMA1|DCTN4|RPL32|LAMTOR3|RPL28
Metabolism of proteins	KLHL20|MRPL51|FBXO30|TFG|CCDC59|SMURF2|ARFGAP3|NOP58|CUL3|ERCC8|FN3KRP|RPS11|PSMA5|FEM1C|CCT8|MRPL39|ALG10B|FARS2|USP9X|MIA3|MRPL20|PPA2|RPS17|RPL22|AP3M1|PSMD12|SIAH1|MANEA|CTR9

	|EPRS|MDM4|CDC73|RNF2|UAP1|ATF6|RARS2|EXTL2|RPL5|NDC1|RPS4X|RPL10A|STAG1|DCP2|RPL24|RPL14|SOCS6|RPL36A|PSMA1|DCTN4|RPL32|SRP19|RPL28|SEC22B|FBXW2|BIRC2
Processing of Intronless Pre-mRNAs	PAPOLA|CSTF3|WDR33|NCBP1
