# Supplementary material for: Meta-Analysis of RNA-Seq Datasets Identifies Novel Players in Glioblastoma
Source: Cancers (Basel). 2022 Nov 24;14(23):5788. doi: 10.3390/cancers14235788 (PMC9737249; doi:10.3390/cancers14235788)
Supplement: Supplementary file 1 [file cancers-14-05788-s001.zip › supplementary file S2/DEPCG PPI network according to Reactome pathway.html]

AAMDCABHD17AACO1ADNPAGO3AGPSAHCTF1ALG10BANKRD10ANKRD50AP1S2AP3M1AQRARFGAP3ARFGEF1ARHGAP15ARID2ASAH1ATF6BCCIPBIRC2BMP2KBMPR1ABRK1BTG2C11orf73CAMK2N2CC2D2ACCDC58CCDC59CCT8CD180CD302CDC23CDC73CEP112CEP83CHD9CNOT10CNTRLCOILCSPP1CSTF3CTR9CUL3CYTH4DCLRE1CDCP2DCTN4DDX46DHX15DHX29DHX32DIAPH2DOCK11DPY30DR1DROSHAEEA1EGR2EIF2AEIF2AK2ELP4EMCNEPRSERCC8EXTL2FARS2FBXW2FEM1CFGF2FNBP1LFNBP4FNDC3AFUBP1FYBGAMTGKGPATCH1GYG1HEATR6HPS5INTS4INTS7INTS8INVSIPO11IPO8IQCA1JADE3KDM7AKIAA1033KIAA1919KPNA4L3HYPDHLAMTOR3LARP7LEPROTLIPT1LSM3LUC7LM6PRMAKMANEAMAP3K27-MarMDM4MED13MED13LMETAP1DMETAP2METTL3MIA3MRE11AMRPL20MRPL39MRPL51NACANBEAL1NCBP1NCOA3NDC1NOP58NSMAFOTUD4PAPOLAPARP11PCBD2PDZD11PHACTR4PHKA1PIK3AP1PIK3CAPINX1PLEKHF2PNRC2POLIPOLR1APOLR2BPOLR3CPPT1PRPF38BPSMA1PSMA5PSMD12PSMD6PTAR1PTBP3QSER1QTRTD1RAB3IPRARS2RBM27REV3LRHOQRINT1RNF169RNF180RNF2RPGRRPL10ARPL14RPL22RPL24RPL28RPL32RPL36ARPL5RPS11RPS17RPS4XRSRC2SEC22BSF3B1SKILSLC19A2SLC25A51SLC33A1SLC35A3SMG7SMURF2SNAP23SRP19SRSF7SSBSTX2SUB1SYAP1TECPR2TET2TFB1MTFGTFRCTGDSTRAPPC11TRIP12U2SURPUBR5UFC1USP9XUTP11LUTP15WDR36YIPF4YIPF6ZNF254ZNF532ZNF566SMCHD1SLC25A17SUPT20HSTT3BPACSIN2PRKAR1ASMPDL3ARPS6KB1WDR75DENND4CNCKAP1LFAM131ARAB3GAP2GBASZNF75DWDR33TLR1RPAP3HSPB11RBM45ZNF277NPATZNF614KLHL20VPRBPWNK3ZNF780AGCNT2FAM118BROCK1MTBPIFNAR1UNC50THUMPD2MMP16ORMDL1SARDHZWILCHULK4ZNF608PPA2ZNF81SCYL2ZNF2MFSD1PKN2STK38LZCCHC10TRAPPC2P1TM7SF2TIMM9ZNF805SOCS6ZNF253TMEM154ZNF512SLC35F5UAP1ZUFSPZNF460ZNF85
