# Supplementary material for: Meta-Analysis of RNA-Seq Datasets Identifies Novel Players in Glioblastoma
Source: Cancers (Basel). 2022 Nov 24;14(23):5788. doi: 10.3390/cancers14235788 (PMC9737249; doi:10.3390/cancers14235788)
Supplement: Supplementary file 1 [file cancers-14-05788-s001.zip › Supplementary file S3.pdf]

Supplementary table S1: Available literature-based evidence of glioblastoma functional associations of the DANCR-targeted DEPCG overlapping with TCGA-GBM

DEPCG	Association with GBM	Reference
ROCK1<br><br>Rho-associated kinase 1	Knockdown induced antidromic cell migration and reduced proliferation<br><br>Inhibition by miR-145 decreased cell invasiveness<br><br>Inhibition blocked macrophage migration inhibitory factor (MIF)- mediated increase in migration and colony formation<br><br>Inhibition by miR-206 overexpression led to inhibition of migration, invasion, and PI3K/AKT pathway activation<br><br>Promotion of migration, invasion and proliferation	[1]<br><br>[2]<br><br>[3]<br><br>[4]<br><br>[5]
GK<br><br>Glycerol kinase	Predicted to act as downstream transcription factor in COL5A1 regulation of cell mobility, metastasis and actin polimerization status	[6]
METAP2<br><br>Methionine aminopeptidase 2	Knockdown decreased proliferation, tumorigeneicity, decreased VEGF expression and dependent angiogenesis	[7]
CIP2A<br><br>Cancerous inhibitor of protein phosphatase 2A	Inhibition induced cell senescence and retarded tumor growth<br><br>Silencing enhanced Cucurbitacin B-induced invasion inhibition and apoptosis<br><br>Overexpression reversed cell cycle and apoptotic protein expression led by anti-tumor 2,5-Dimethyl Celecoxib<br><br>Promotion of viability, clonogenicity and anchorage-independent growth	[8]<br><br>[9]<br><br>[10]<br><br>[11]
ASAH1<br><br>Acid ceramidase	Expression associated with poor survival and inhibition increases cellular ceramide level and induces apoptosis<br><br>Upregulation conferred cellular radioresistance	[12]<br><br>[13]
STX2<br><br>Syntaxin 2	Inhibition reduced growth of tumor xenografts in vivo	[14]

MAP3K2<br><br>Mitogen-activated protein kinase 2	Restoration of proliferation occurring due to circ-PITX1 silencing	[15]

Supplementary figure S1: Functional enrichment of DEmiRNAs performed using g:Profiler

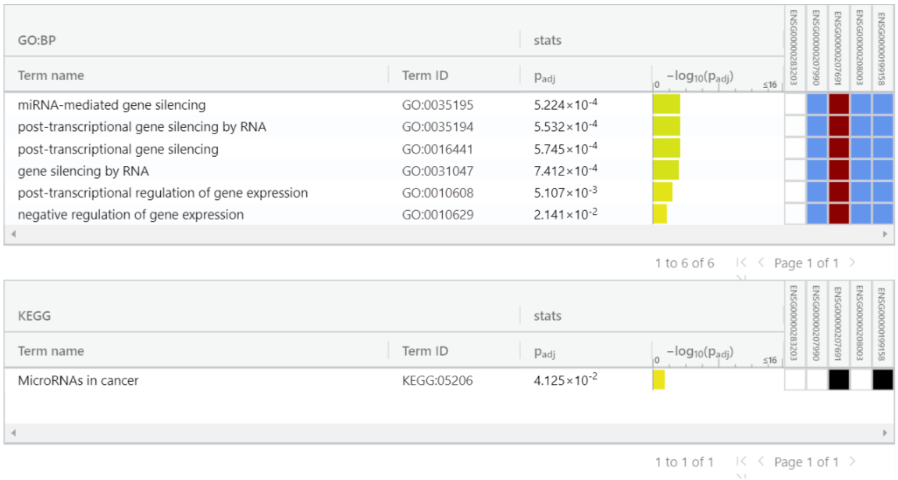

Supplementary figure S2: Co-expression network of DElncRNA/DEPCG co-expression correlation network constructed using Cytoscape's Metscape App and showing only pairs with Pearson correlation coefficient (r) > |0.7| with $p < 0.05$ (***double click to enlarge***)

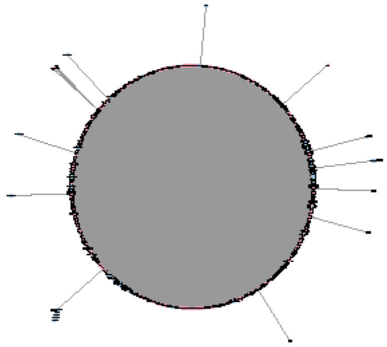

Supplementary figure S3: Visualization of the top four clusters (A,B,C and D, respectively) containing ≥10 members, obtained by analyzing co- expression correlation between DElncRNAs and DEPCGs using MCODE in Cytoscape. The first cluster was further analyzed using MCODE to produce sub-clusters of which the 3 top sub-clusters were visualized (A.1, A.2 and A.3 respectively). Blue triangles and circles indicate DElncRNAs and pink circles indicate DEPCGs. The thickness of a line indicates the strength of the interaction between the proteins it connects.

A) Cluster 1 reclustered using *MCODE*

Cluster A.1

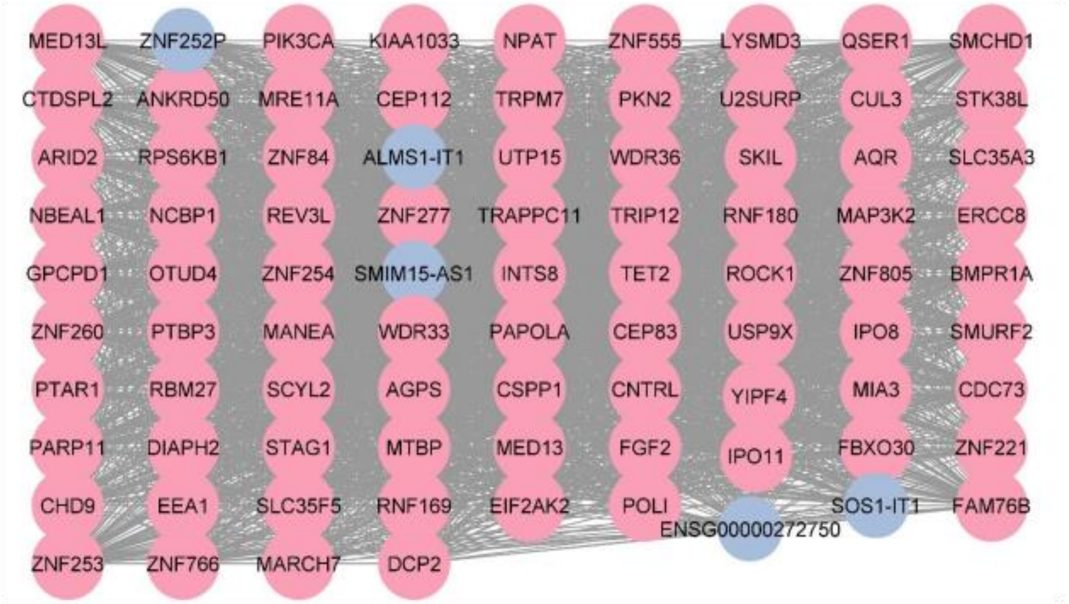

Cluster A.2

Figure 4A in manuscript

Cluster A.3

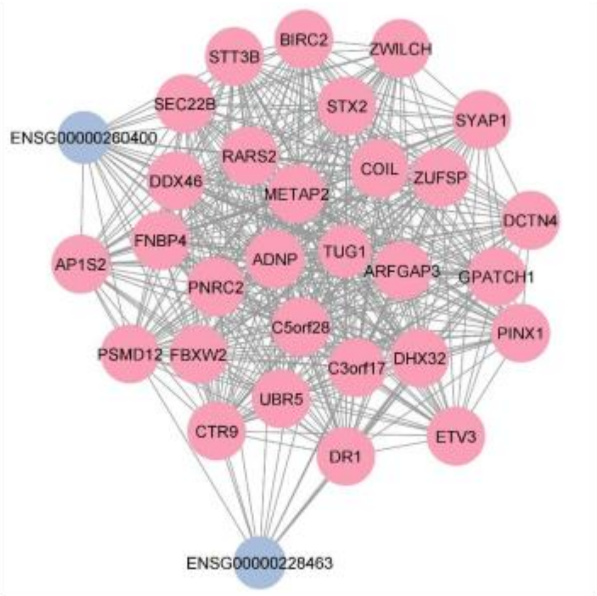

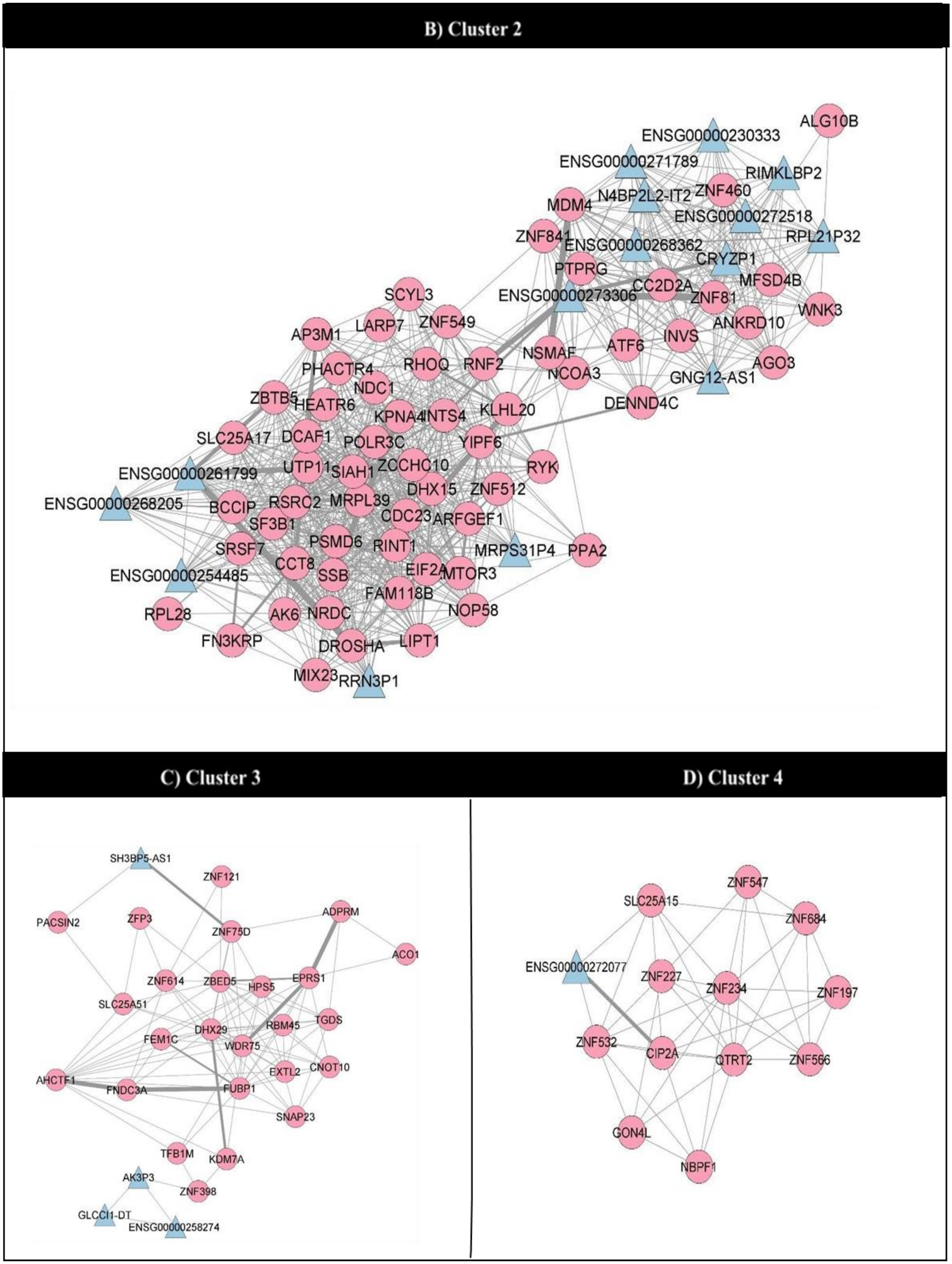
B) Cluster 2
ALG10B
ENSG00000230333
ENSG00000271789
RIMKLBP2
N4BP2L2-IT2
ZNF460
MDM4
ENSG00000272518
ZNF841
ENSG00000268362
RPL21P32
CRYZP1
PTPRG
MFSD4B
SCYL3
CC2D2A
ENSG00000273306
ZNF81
WNK3
AP3M1
LARP7
ZNF549
ANKRD10
INVS
ATF6
NSMAF
AGO3
PHACTR4
RHOQ
RNF2
NCOA3
GNG12-AS1
NDC1
ZBTB5
HEATR6
KLHL20
DENND4C
KPNA4
INTS4
SLC25A17
DCAF1
POLR3C
YIPF6
ENSG00000261799
UTP11
SIAH1
ZCCHC10
RYK
DHX15
ZNF512
ENSG00000268205
BCCIP
RSRC2
MRPL39
SF3B1
CDC23
ARFGEF1
PSMD6
RINT1
MRPS31P4
PPA2
SRSF7
CCT8
EIF2A
MTOR3
ENSG00000254485
SSB
FAM118B
NOP58
RPL28
AK6
NRDC
FN3KRP
DROSHA
LIPT1
MIX23
RRN3P1
C) Cluster 3
SH3BP5-AS1
ZNF121
PACSIN2
ZFP3
ZNF75D
ADPRM
ACO1
ZNF614
ZBED5
HPS5
EPRS1
SLC25A51
TGDS
DHX29
RBM45
FEM1C
WDR75
EXTL2
CNOT10
AHCTF1
FNDC3A
FUBP1
SNAP23
TFB1M
KDM7A
AK3P3
ZNF398
GLCCI1-DT
ENSG00000258274
D) Cluster 4
ZNF547
SLC25A15
ZNF684
ENSG00000272077
ZNF227
ZNF234
ZNF197
ZNF532
CIP2A
QTRT2
ZNF566
GON4L
NBPF1

Supplementary table S2: Available literature-based evidence of glioblastoma/ferroptosis functional associations of DEPCGs in the DANCR/SNHG6 sub-cluster of co-expression correlation network

	**Characterized role in glioma/glioblastoma**	**previously associated with ferroptosis**
LUC7L	Yes [16]	No
PDZD11	No	No
BRK1	No	No
UFC1	No	Part of UFMylation pathway which regulates ferroptosis in breast cancer [17]
ENSG00000256646	No	No
TAF15	Predicted target of LINC01564 which promotes glioma cell treatment resistance [18]	Predicted target of LINC01564 which inhibits ferroptosis [18]
ALDH9A1	Activated by CLOCK which drives immunosuppression in glioblastoma [19,20]	No
TIMM9	By machine learning from high throughput CRISPR-Cas9 [21]	No
MRPL20	No	No
RPS4X	Part of in-silico glioblastoma prognostic model [22]	No
RPL36A	Part of prognostic model for GBM (Preprint [22], [23]	No
PRPF38B	No	No
ZNF266	No	No
MRPL51	No	No
RPL5	No	No
PSMA5	Independent prognostic marker for glioma and combined treatment with carboplatin and thioridazine was shown to induce apoptosis by upregulation of Nrf2-dependent PSMA5 expression [24,25].	No
SELK	Regulates proliferation, drug sensitivity and invasion of glioma cells [26].	Induced by Selenium which drives ferroptosis inhibition [27]
ORMDL1	No	No
RPL24	No	No
RPL32	No	No
PPT1	elevated expression of PPT1 correlates with poor survival in TCGA patients with gliomas [28]	No
ENSG00000255639	No	No
RPL14	No	No

PSMA1	Identified during a screen for genes contributing to radiation and temozolamide sensitivity as well as tumoricidal activity	Induced by NRF2 which regulates ferroptosis [29–32]
C11orf73	No	Mediates nuclear translocation of heat shock protein 70 (HSP70) which regulates ferroptosis [33,34]
METTL3	Regulates the proliferation, migration and invasion of glioma cells [35]	Regulates ferroptosis [36,37]
ZNF547	No	No
HSPB11	inhibits cell death by HSP90 mediated mechanism [38]prognostic marker of high grade glioma [39]	No
DPY30	Drives glioblastoma growth in vivo [40]	No
SRP19	No	No
RPAIN	No	No
MITD1	No	Deficiency induces renal carcinoma growth and migration by ferroptosis induction [41]
UNC50	No	No
IFNGR2	Identified in glioblastoma by genome-wide CRISPR screen [42] and may serve as a biomarker to stratify glioblastoma patients responsiveness to immune checkpoint blockade based therapies [43]	No
NACA	No	No
RPL22	No	No
NDUFA9	No	No
M6PR	No	No
CBWD1 or ZNG1A	No	No
SUB1	Enhances proliferation and migration of glioma cells [44]	No
LSM3	No	No
RPL10A	No	No
SMG7	No	Smg7 $^{-/-}$ cells showed increased protection against cell death by ferroptosis-inducer Erastin [45]
C5orf15	No	No

TFG	Fusion with MET with overexpression of TFG-MET induces aggressive glial brain tumors in Cdkn2a- or Trp53-deficient mice [46].	No
RPS11	influences glioma response to TOP2 poisons [47]<br>predictor of poor prognosis in glioma [48]	No
GYG1	No	No
RAB3GAP2	No	No
THUMPD2	Isoform changes regulate glioma cell line sensitivity to temozolomide [49]	No
SUPT20H	Part of prognostic risk score model for TCGA and CGGA gliomas [50]	No
CCDC59 or TAP26	No	No

Supplementary figure S4: Chinese Glioma Genome Atlas (CCGA) Pearson correlation analysis performed using the ‘Analyze’ tab in CCGA portal (http://www.cgga.org.cn/) between DANCR/SNHG6 in mRNAseq 693 (A) and mRNAseq 325 (B) and between miR96/miR182 (C), miR96/miR183 (D) and miR182-miR183 (E) in microRNA_array_198 of CCGA.

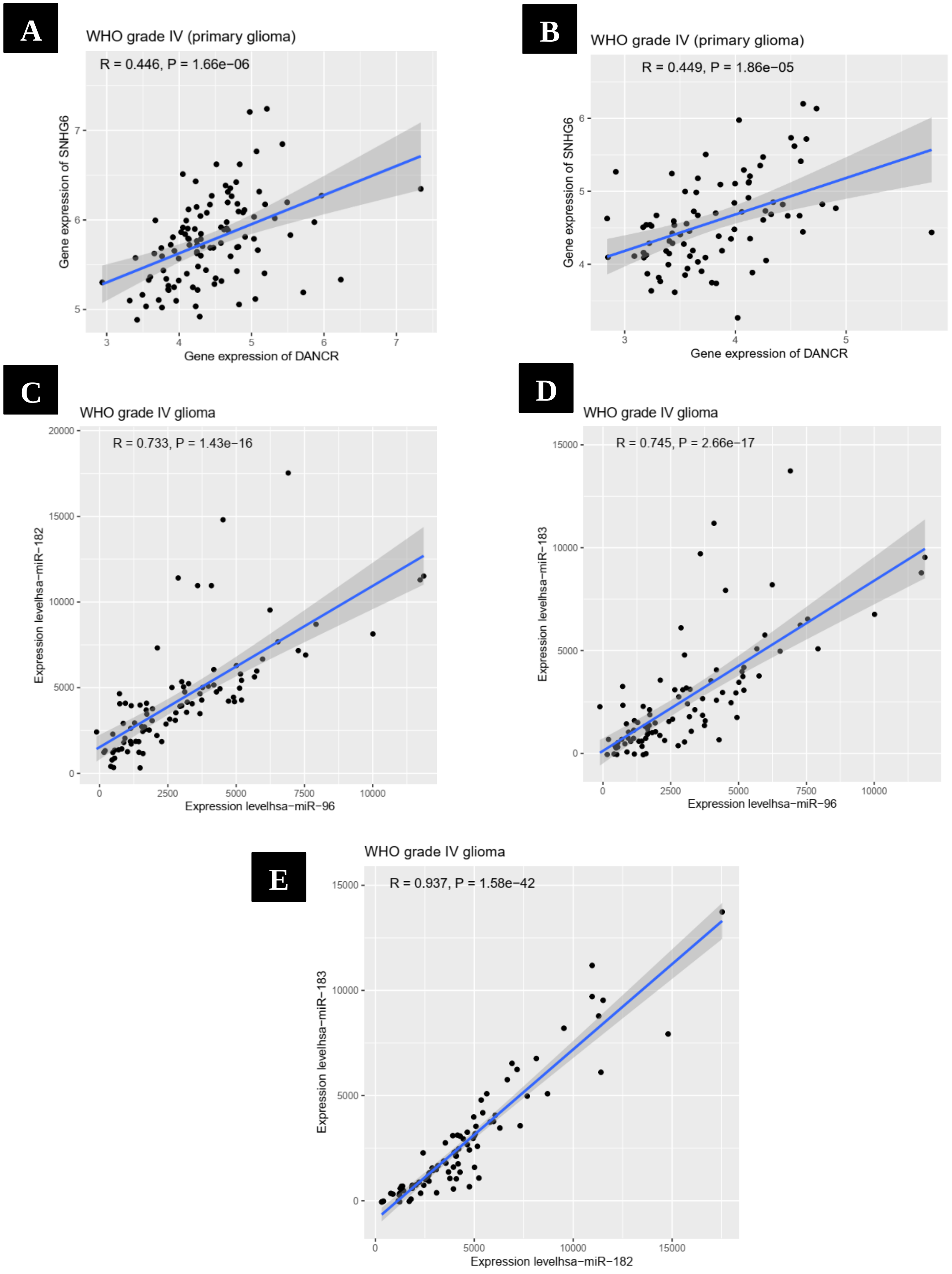

Supplementary references:

1. Mertsch, S.; Thanos, S. Opposing Signaling of ROCK1 and ROCK2 Determines the Switching of Substrate Specificity and the Mode of Migration of Glioblastoma Cells. *Mol. Neurobiol.* **2014**, *49*, 900–915, doi:10.1007/S12035-013-8568-6/FIGURES/8.

2. Wan, X.; Cheng, Q.; Peng, R.; Ma, Z.; Chen, Z.; Cao, Y.; Jiang, B. ROCK1, a Novel Target of MiR-145, Promotes Glioma Cell Invasion. *Mol. Med. Rep.* **2014**, *9*, 1877–1882, doi:10.3892/MMR.2014.1982/HTML.

3. Xu, S.; Guo, X.; Gao, X.; Xue, H.; Zhang, J.; Guo, X.; Qiu, W.; Zhang, P.; Li, G. Macrophage Migration Inhibitory Factor Enhances Autophagy by Regulating ROCK1 Activity and Contributes to the Escape of Dendritic Cell Surveillance in Glioblastoma. *Int. J. Oncol.* **2016**, *49*, 2105–2115, doi:10.3892/IJO.2016.3704/HTML.

4. Wang, D.; Yang, T.; Liu, J.; Liu, Y.; Xing, N.; He, J.; Yang, J.; Ai, Y. Propofol Inhibits the Migration and Invasion of Glioma Cells by Blocking the PI3K/AKT Pathway Through MiR-206/ROCK1 Axis. *Onco. Targets. Ther.* **2020**, *13*, 361, doi:10.2147/OTT.S232601.

5. Chen, X.; Li, D.; Chen, L.; Hao, B.; Gao, Y.; Li, L.; Zhou, C.; He, X.; Cao, Y. Long Noncoding RNA LINC00346 Promotes Glioma Cell Migration, Invasion and Proliferation by up-Regulating ROCK1. *J. Cell. Mol. Med.* **2020**, *24*, 13010–13019, doi:10.1111/JCMM.15899.

6. Tsai, H.F.; Chang, Y.C.; Li, C.H.; Chan, M.H.; Chen, C.L.; Tsai, W.C.; Hsiao, M. Type V Collagen Alpha 1 Chain Promotes the Malignancy of Glioblastoma through PPRC1-ESM1 Axis Activation and Extracellular Matrix Remodeling. *Cell Death Discov. 2021 71* **2021**, *7*, 1–12, doi:10.1038/s41420-021-00661-3.

7. Lin, M.; Zhang, X.; Jia, B.; Guan, S. Suppression of Glioblastoma Growth and Angiogenesis through Molecular Targeting of Methionine Aminopeptidase-2. *J. Neurooncol.* **2018**, *136*, 243–254, doi:10.1007/S11060-017-2663-X/FIGURES/6.

8. Khanna, A.; Thoms, J.A.I.; Stringer, B.W.; Chung, S.A.; Ensbey, K.S.; Jue, T.R.; Jahan, Z.; Subramanian, S.; Anande, G.; Shen, H.; et al. Constitutive CHK1 Expression Drives a PSTAT3-CIP2A Circuit That Promotes Glioblastoma Cell Survival and Growth. *Mol. Cancer Res.* **2020**, *18*, 709–722, doi:10.1158/1541-7786.MCR-19-0934/82151/AM/CONSTITUTIVE-CHK1-EXPRESSION-DRIVES-A-PSTAT3-CIP2A.

9. Qin, S.; Li, J.; Si, Y.; He, Z.; Zhang, T.; Wang, D.; Liu, X.; Guo, Y.; Zhang, L.; Li, S.; et al. Cucurbitacin B Induces Inhibitory Effects via CIP2A/PP2A/Akt Pathway in Glioblastoma Multiforme. *Mol. Carcinog.* **2018**, *57*, 687–699, doi:10.1002/MC.22789.

10. Gao, D.; Nyalali, A.M.K.; Hou, Y.; Xu, Y.; Zhou, J.; Zhao, W.; Huang, B.; Li, F. 2,5-Dimethyl Celecoxib Inhibits Proliferation and Cell Cycle and Induces Apoptosis in Glioblastoma by Suppressing CIP2A/PP2A/Akt Signaling Axis. *J. Mol. Neurosci.* **2021**, *71*, 1703–1713, doi:10.1007/S12031-020-01773-8/FIGURES/5.

11. Khanna, A.; Stringer, B.; Day, B.; Ensbey, K.; Shen, H.; Boyd, A.; McDonald, K.; Pimanda, J.E. Abstract 1600: Keeping Glioblastoma (GBM)in Check by Targeting the CHK1-STAT3-CIP2A Axis. *Cancer Res.* **2014**, *74*, 1600–1600, doi:10.1158/1538-

7445.AM2014-1600.

12. Doan, N.B.; Alhajala, H.; Al-Gizawiy, M.M.; Mueller, W.M.; Rand, S.D.; Connelly, J.M.; Cochran, E.J.; Chitambar, C.R.; Clark, P.; Kuo, J.; et al. Acid Ceramidase and Its Inhibitors: A de Novo Drug Target and a New Class of Drugs for Killing Glioblastoma Cancer Stem Cells with High Efficiency. *Oncotarget* **2017**, *8*, 112662, doi:10.18632/ONCOTARGET.22637.

13. Doan, N.B.; Nguyen, H.S.; Al-Gizawiy, M.M.; Mueller, W.M.; Sabbadini, R.A.; Rand, S.D.; Connelly, J.M.; Chitambar, C.R.; Schmainda, K.M.; Mirza, S.P. Acid Ceramidase Confers Radioresistance to Glioblastoma Cells. *Oncol. Rep.* **2017**, *38*, 1932–1940, doi:10.3892/OR.2017.5855/HTML.

14. Ulloa, F.; Gonzàlez-Juncà, A.; Meffre, D.; Barrecheguren, P.J.; Martínez-Mármol, R.; Pazos, I.; Olivé, N.; Cotrufo, T.; Seoane, J.; Soriano, E. Blockade of the SNARE Protein Syntaxin 1 Inhibits Glioblastoma Tumor Growth. *PLoS One* **2015**, *10*, e0119707, doi:10.1371/JOURNAL.PONE.0119707.

15. Lv, X.; Wang, M.; Qiang, J.; Guo, S. Circular RNA Circ-PITX1 Promotes the Progression of Glioblastoma by Acting as a Competing Endogenous RNA to Regulate MiR-379–5p/MAP3K2 Axis. *Eur. J. Pharmacol.* **2019**, *863*, 172643, doi:10.1016/J.EJPHAR.2019.172643.

16. Pan, Y.-B.; Wang, S.; Yang, B.; Jiang, Z.; Lenahan, C.; Wang, J.; Zhang, J.; Shao, A. Transcriptome Analyses Reveal Molecular Mechanisms Underlying Phenotypic Differences among Transcriptional Subtypes of Glioblastoma. *J. Cell. Mol. Med.* **2020**, *24*, 3901–3916, doi:https://doi.org/10.1111/jcmm.14976.

17. Yang, J.; Zhou, Y.; Xie, S.; Wang, J.; Li, Z.; Chen, L.; Mao, M.; Chen, C.; Huang, A.; Chen, Y.; et al. Metformin Induces Ferroptosis by Inhibiting UFMylation of SLC7A11 in Breast Cancer. *J. Exp. Clin. Cancer Res.* **2021**, *40*, 206, doi:10.1186/s13046-021-02012-7.

18. Luo, C.; Nie, C.; Zeng, Y.; Qian, K.; Li, X.; Wang, X. LINC01564 Promotes the TMZ Resistance of Glioma Cells by Upregulating NFE2L2 Expression to Inhibit Ferroptosis. *Mol. Neurobiol.* **2022**, *59*, 3829–3844, doi:10.1007/s12035-022-02736-3.

19. Chen, P.; Hsu, W.-H.; Chang, A.; Tan, Z.; Lan, Z.; Zhou, A.; Spring, D.J.; Lang, F.F.; Wang, Y.A.; DePinho, R.A. Circadian Regulator CLOCK Recruits Immune-Suppressive Microglia into the GBM Tumor MicroenvironmentCLOCK in Tumor Immunity. *Cancer Discov.* **2020**, *10*, 371–381.

20. Xuan, W.; Hsu, W.-H.; Khan, F.; Dunterman, M.; Pang, L.; Wainwright, D.A.; Ahmed, A.U.; Heimberger, A.B.; Lesniak, M.S.; Chen, P. Circadian Regulator CLOCK Drives Immunosuppression in Glioblastoma. *Cancer Immunol. Res.* **2022**, *10*, 770–784, doi:10.1158/2326-6066.CIR-21-0559.

21. Xiang, C.; Liu, X.; Zhou, D.; Zhou, Y.; Wang, X.; Chen, F. Identification of a Glioma Functional Network from Gene Fitness Data Using Machine Learning. *J. Cell. Mol. Med.* **2022**, *26*, 1253–1263, doi:https://doi.org/10.1111/jcmm.17182.

22. Zhao, S.; Ji, W.; Shen, Y.; Fan, Y.; Huang, J.; Huang, H.; Cheng, C.; Shao, J. Expression

of Hub Genes of Endothelial Cells in Glioblastoma-A Prognostic Model for GBM Patients Integrating Single Cell RNA Sequencing and Bulk RNA Sequencing 2022.

23. Li, R.; Jiang, Q.; Tang, C.; Chen, L.; Kong, D.; Zou, C.; Lin, Y.; Luo, J.; Zou, D. Identification of Candidate Genes Associated With Prognosis in Glioblastoma . *Front. Mol. Neurosci.* 2022, *15*.

24. Gao, F.; Wang, Z.; Gu, J.; Zhang, X.; Wang, H. A Hypoxia-Associated Prognostic Gene Signature Risk Model and Prognosis Predictors in Gliomas . *Front. Oncol.* 2021, *11*.

25. Seo, S.U.; Cho, H.K.; Min, K.; Woo, S.M.; Kim, S.; Park, J.-W.; Kim, S.H.; Choi, Y.H.; Keum, Y.S.; Hyun, J.W.; et al. Thioridazine Enhances Sensitivity to Carboplatin in Human Head and Neck Cancer Cells through Downregulation of C-FLIP and Mcl-1 Expression. *Cell Death Dis.* **2017**, *8*, e2599–e2599, doi:10.1038/cddis.2017.8.

26. Xu, C.-H.; Xiao, L.-M.; Zeng, E.-M.; Chen, L.-K.; Zheng, S.-Y.; Li, D.-H.; Liu, Y. MicroRNA-181 Inhibits the Proliferation, Drug Sensitivity and Invasion of Human Glioma Cells by Targeting Selenoprotein K (SELK). *Am. J. Transl. Res.* **2019**, *11*, 6632.

27. Alim, I.; Caulfield, J.T.; Chen, Y.; Swarup, V.; Geschwind, D.H.; Ivanova, E.; Seravalli, J.; Ai, Y.; Sansing, L.H.; Ste.Marie, E.J.; et al. Selenium Drives a Transcriptional Adaptive Program to Block Ferroptosis and Treat Stroke. *Cell* **2019**, *177*, 1262-1279.e25, doi:https://doi.org/10.1016/j.cell.2019.03.032.

28. Tang, F.; Liu, Z.; Chen, X.; Yang, J.; Wang, Z.; Li, Z. Current Knowledge of Protein Palmitoylation in Gliomas. *Mol. Biol. Rep.* **2022**, doi:10.1007/s11033-022-07809-z.

29. Li, J.; Zhang, M.; An, G.; Ma, Q. LncRNA TUG1 Acts as a Tumor Suppressor in Human Glioma by Promoting Cell Apoptosis. *Exp. Biol. Med. (Maywood).* **2016**, *241*, 644–649, doi:10.1177/1535370215622708.

30. Zhao, Y.; Lu, J.; Liu, M.; Guan, S. Toward Improved Human Health: Nrf2 Plays a Critical Role in Regulating Ferroptosis. *Food Funct.* **2021**.

31. Song, X.; Long, D. Nrf2 and Ferroptosis: A New Research Direction for Neurodegenerative Diseases. *Front. Neurosci.* **2020**, *14*, 267.

32. Torrente, L.; DeNicola, G.M. Targeting NRF2 and Its Downstream Processes: Opportunities and Challenges. *Annu Rev Pharmacol Toxicol* **2022**, *62*, 279–300.

33. Liu, Y.; Zhou, L.; Xu, Y.; Li, K.; Zhao, Y.; Qiao, H.; Xu, Q.; Zhao, J. Heat Shock Proteins and Ferroptosis . *Front. Cell Dev. Biol.* 2022, *10*.

34. Yanoma, T.; Ogata, K.; Yokobori, T.; Ide, M.; Mochiki, E.; Toyomasu, Y.; Yanai, M.; Kogure, N.; Kimura, A.; Suzuki, M.; et al. Heat Shock-Induced HIKESHI Protects Cell Viability via Nuclear Translocation of Heat Shock Protein 70 . *Oncol Rep* **2017**, *38*, 1500–1506, doi:10.3892/or.2017.5844.

35. Ji, J.-W.; Zhang, Y.-D.; Lai, Y.-J.; Huang, C.-G. Mettl3 Regulates the Proliferation, Migration and Invasion of Glioma Cells by Inhibiting PI3K/Akt Signaling Pathway. *Eur. Rev. Med. Pharmacol. Sci.* **2020**, *24*, 3818–3828, doi:10.26355/eurrev_202004_20848.

36. Xu, Y.; Lv, D.; Yan, C.; Su, H.; Zhang, X.; Shi, Y.; Ying, K. METTL3 Promotes Lung

Adenocarcinoma Tumor Growth and Inhibits Ferroptosis by Stabilizing SLC7A11 M6A Modification. *Cancer Cell Int.* **2022**, *22*, 11, doi:10.1186/s12935-021-02433-6.

37. Li, N.; Yi, X.; He, Y.; Huo, B.; Chen, Y.; Zhang, Z.; Wang, Q.; Li, Y.; Zhong, X.; Li, R. Targeting Ferroptosis as a Novel Approach to Alleviate Aortic Dissection. *Int. J. Biol. Sci.* **2022**, *18*, 4118–4134.

38. Turi, Z.; Hocsak, E.; Racz, B.; Szabo, A.; Balogh, A.; Sumegi, B.; Gallyas Jr, F. Role of Mitochondrial Network Stabilisation by a Human Small Heat Shock Protein in Tumour Malignancy. *J. Cancer* **2015**, *6*, 470.

39. Cheng, W.; Li, M.; Jiang, Y.; Zhang, C.; Cai, J.; Wang, K.; Wu, A. Association between Small Heat Shock Protein B11 and the Prognostic Value of MGMT Promoter Methylation in Patients with High-Grade Glioma. *J. Neurosurg.* **2016**, *125*, 7–16.

40. Dixit, D.; Prager, B.C.; Gimple, R.C.; Miller, T.E.; Wu, Q.; Yomtoubian, S.; Kidwell, R.L.; Lv, D.; Zhao, L.; Qiu, Z.; et al. Glioblastoma Stem Cells Reprogram Chromatin in Vivo to Generate Selective Therapeutic Dependencies on DPY30 and Phosphodiesterases. *Sci. Transl. Med.* **2022**, *14*, eabf3917, doi:10.1126/scitranslmed.abf3917.

41. Zhang, Y.; Li, Y.; Qiu, Q.; Chen, Z.; Du, Y.; Liu, X. MITD1 Deficiency Suppresses Clear Cell Renal Cell Carcinoma Growth and Migration by Inducing Ferroptosis through the TAZ/SLC7A11 Pathway. *Oxid. Med. Cell. Longev.* **2022**, *2022*, 7560569, doi:10.1155/2022/7560569.

42. Hong, L.; Ye, L. The Interferon-γ Receptor Pathway: A New Way to Regulate CAR T Cell-Solid Tumor Cell Adhesion. *Signal Transduct. Target. Ther.* **2022**, *7*, 315, doi:10.1038/s41392-022-01165-x.

43. Chen, D.; Varanasi, S.K.; Hara, T.; Traina, K.; McDonald, B.; Farsakoglu, Y.; Clanton, J.; Xu, S.; Mann, T.H.; Du, V.; et al. A Microglia-CD4+ T Cell Partnership Generates Protective Anti-Tumor Immunity to Glioblastoma. *bioRxiv* **2022**, 2022.08.12.502093, doi:10.1101/2022.08.12.502093.

44. Liu, Z.; Wang, J.; Wang, J.; Niu, J.; Wang, J.; Tong, H. CircVCAN/SUB1 up-Regulates MYC/HSP90β to Enhance the Proliferation and Migration of Glioma Cells. *Brain Res. Bull.* **2021**, *177*, 332–339.

45. Trümbach, D.; Pfeiffer, S.; Poppe, M.; Scherb, H.; Doll, S.; Wurst, W.; Schick, J.A. ENCoRE: An Efficient Software for CRISPR Screens Identifies New Players in Extrinsic Apoptosis. *BMC Genomics* **2017**, *18*, 905, doi:10.1186/s12864-017-4285-2.

46. Bender, S.; Gronych, J.; Warnatz, H.-J.; Hutter, B.; Gröbner, S.; Ryzhova, M.; Pfaff, E.; Hovestadt, V.; Weinberg, F.; Halbach, S.; et al. Recurrent MET Fusion Genes Represent a Drug Target in Pediatric Glioblastoma. *Nat. Med.* **2016**, *22*, 1314–1320, doi:10.1038/nm.4204.

47. Awah, C.U.; Chen, L.; Bansal, M.; Mahajan, A.; Winter, J.; Lad, M.; Warnke, L.; Gonzalez-Buendia, E.; Park, C.; Zhang, D. Ribosomal Protein S11 Influences Glioma Response to TOP2 Poisons. *Oncogene* **2020**, *39*, 5068–5081.

48. Yong, W.H.; Shabihkhani, M.; Telesca, D.; Yang, S.; Tso, J.L.; Menjivar, J.C.; Wei, B.;

Lucey, G.M.; Mareninov, S.; Chen, Z.; et al. Ribosomal Proteins RPS11 and RPS20, Two Stress-Response Markers of Glioblastoma Stem Cells, Are Novel Predictors of Poor Prognosis in Glioblastoma Patients. *PLoS One* **2015**, *10*, e0141334.

49. Tiek, D.M.; Erdogdu, B.; Razaghi, R.; Jin, L.; Sadowski, N.; Alamillo-Ferrer, C.; Hogg, J.R.; Haddad, B.R.; Drewry, D.H.; Wells, C.I.; et al. Temozolomide-Induced Guanine Mutations Create Exploitable Vulnerabilities of Guanine-Rich DNA and RNA Regions in Drug-Resistant Gliomas. *Sci. Adv.* **2022**, *8*, eabn3471, doi:10.1126/sciadv.abn3471.

50. Xu, Y.; Li, R.; Li, X.; Dong, N.; Wu, D.; Hou, L.; Yin, K.; Zhao, C. An Autophagy-Related Gene Signature Associated With Clinical Prognosis and Immune Microenvironment in Gliomas . *Front. Oncol.* 2020, *10*.
